# Rapamycin Treatment Reduces Brain Pericyte Constriction in Ischemic Stroke

**DOI:** 10.1007/s12975-024-01298-x

**Published:** 2024-09-27

**Authors:** Daniel J. Beard, Lachlan S. Brown, Gary P. Morris, Yvonne Couch, Bryan A. Adriaanse, Christina Simoglou Karali, Anna M. Schneider, David W. Howells, Zoran B. Redzic, Brad A. Sutherland, Alastair M. Buchan

**Affiliations:** 1 Present Address: Acute Stroke Programme, Radcliffe Department of Medicine, University of Oxford, Oxford, UK; 2 School of Biomedical Sciences and Pharmacy, University of Newcastle, Newcastle, Australia; 3 Tasmanian School of Medicine, College of Health and Medicine, University of Tasmania, Hobart, Australia; 4 Nuffield Department of Clinical Neurosciences, University of Oxford, Oxford, UK; 5 Department of Oncology, University of Oxford, Oxford, UK; 6 Department of Physiology, Faculty of Medicine, Kuwait University, Kuwait City, Kuwait

**Keywords:** Brain pericytes, Experimental stroke, Rapamycin, mTOR, Calcium

## Abstract

The contraction and subsequent death of brain pericytes may play a role in microvascular no-reflow following the reopening of an occluded artery during ischemic stroke. Mammalian target of rapamycin (mTOR) inhibition has been shown to reduce motility/contractility of various cancer cell lines and reduce neuronal cell death in stroke. However, the effects of mTOR inhibition on brain pericyte contraction and death during ischemia have not yet been investigated. Cultured pericytes exposed to simulated ischemia for 12 h in vitro contracted after less than 1 h, which was about 7 h prior to cell death. Rapamycin significantly reduced the rate of pericyte contraction during ischemia; however, it did not have a significant effect on pericyte viability at any time point. Rapamycin appeared to reduce pericyte contraction through a mechanism that is independent of changes in intracellular calcium. Using a mouse model of middle cerebral artery occlusion, we showed that rapamycin significantly increased the diameter of capillaries underneath pericytes and increased the number of open capillaries 30 min following recanalisation. Our findings suggest that rapamycin may be a useful adjuvant therapeutic to reduce pericyte contraction and improve cerebral reperfusion post-stroke.

**Supplementary information:**

The online version contains supplementary material available at 10.1007/s12975-024-01298-x.

## Introduction

Pericytes are vascular mural cells located between endothelial cells and astrocyte end-feet within the basal lamina of capillaries and are found in particularly high density on capillaries of the central nervous system compared to other organs [1]. Recent evidence suggests that pericytes are contractile and regulate the luminal size of capillaries both in vitro and in vivo [2, 3]. This is particularly relevant in ischemic stroke as irreversible pericyte constriction (‘death in rigour’) has been implicated as a major component of ‘no-reflow’ in preclinical models, a phenomenon where microvascular patency and blood flow are not restored even after large artery occlusions are removed [4]. This reduces the effectiveness of stroke therapies solely focusing on revascularization such as thrombolysis and thrombectomy in model systems [2, 5]. The no-reflow phenomenon also occurs in human stroke with one-third of stroke patients reported to have microvascular perfusion deficits despite successful arterial recanalization [6]. Preventing brain pericyte death in rigour and/or ischemia-triggered contraction is therefore a potential therapeutic target to improve microvascular perfusion post-recanalization [7].

The Stroke Treatment Academic Round Table X (STAIR X) guidelines recommend prioritising cytoprotective approaches that exert pleiotropic effects on multiple targets of the ischemic cascade and protect all brain components affected in stroke [8, 9]. Amplifying the brain’s intrinsic neuroprotective pathways is one such multimodal approach for developing new treatments for stroke. Mammalian target of rapamycin (mTOR) has been shown to play an important role in cell death after stroke [10]. In states of sufficient energy supply, mTORC1 is activated, signalling anabolic cellular processes such as protein synthesis, cell proliferation, cytoskeletal formation, and cellular contractility/motility [10]. Conversely, low cellular energy, i.e. increased AMP to ATP ratio, such as in cerebral ischemia, induces tuberous sclerosis-1 (TSC1, also known as hamartin) and TSC2 (tuberin) to form the TSC complex, subsequently down-regulating mTORC1 [11, 12], which has been shown to be protective in models of brain ischemia [10]. Therefore, enhancement of endogenous mTOR inhibition, and hence, augmentation of cell-preserving effects, is an appealing target for brain cytoprotection in cerebral ischemia.

Rapamycin (Sirolimus™) [13], an immunosuppressant frequently used in humans for prophylaxis of renal transplant rejection and for treatment of lymphangioleiomyomatosis [14], inhibits mTORC1. Inhibition of mTORC1 with rapamycin has been shown to reduce infarct volume and decrease neurological deficits in animal models of cerebral ischemia [15]. Rapamycin can also protect multiple cells within the brain including neurons [16] and brain endothelial cells [17] in in vitro models of ischemia [18]. Rapamycin treatment has also been shown to improve collateral blood flow in experimental stroke by increasing endothelial nitric oxide synthase activity [19]. As rapamycin is already in clinical use with a known side effect profile, potential extrapolation to the stroke clinic is a distinct possibility. In the current study, we investigated if rapamycin could reduce pericyte contractility both in vitro and following experimental stroke *in vivo* and investigated the mechanism by which rapamycin may exert this effect.

## Materials and Methods

### Cell Culture

Primary cultures of pericytes from rat brain were produced using a previously described method from 2- to 3-month-old Wistar rats [20]. This amount of tissue provides enough pericytes for 1 culture. Each subsequent culture was also produced from a set of 5 different rats and plated on different days, making each culture an independent biological replicate (full methodological details in the Supplementary Materials). Human brain vascular pericytes (HBVP) (#1200) were purchased from ScienCell Research Laboratories, cultured in complete pericyte medium, and used between passages 6 and 8.

### Immunocytochemistry

Immunocytochemistry for canonical pericyte markers was performed on primary rat pericytes to confirm the purity of cultures (full methodological details in the Supplementary Materials). We have previously characterised HBVPs extensively [21–23].

### Oxygen Glucose Deprivation

Rat brain pericytes were exposed to either oxygen glucose deprivation (OGD) or to control conditions for 2–12 h. In control experiments, rat brain pericyte cultures were incubated in DMEM (Gibco) that contained a reduced serum concentration (2%), 5 mM glucose, and 1.25 mM pyruvate in 5% CO2 in humidified room air at 37 °C (control/normoxia media). In OGD experiments, cell cultures were incubated in a hypoxic glove box (Coy Laboratories) maintained with 0% O_2_ and 5% CO_2_ in N_2_ at 37 °C. All media and buffers were left in the glove box for at least 12 h prior to OGD experiments to equilibrate partial pressures of gases. When transferred to the glove box, cell cultures were incubated in the medium that had the same composition as for the control experiments, but did not contain glucose and pyruvate (OGD media) (Gibco). The same control and OGD conditions were used for pericyte contractility, western blotting, and cell death analysis.

### Measurement of Rat Pericyte Contractility

We used an electrical impedance system to detect changes in the contact area between rat pericytes and the culture dish, as described previously by Neuhaus et al. [23]. In brief, rat pericytes were plated on impedance plates (E-Plate L8 PET; ACEA Biosciences) and allowed to proliferate in the iCELLigence platform (ACEA Biosciences) for 48 h prior to testing. Pericytes were then exposed to OGD for 12 h and treated with vehicle (0.005% ethanol in media), 10 nM rapamycin (Sigma #553210), or 100 nM rapamycin. The same vehicle and drug concentrations were used for all subsequent experiments. Data are presented as normalised cell index (a unitless parameter, automatically derived from recorded impedance values in iCELLigence software and normalised to the last time point pre-OGD), with the rate of contraction calculated by the slope of the cell index.

### Measurement of Rat Pericyte Cell Death

Flow cytometry was used to measure apoptosis/necrosis in rat pericytes after exposure to 2, 8, or 12 h of OGD or normoxia as well as treatments with vehicle, 10 nM rapamycin, or 100 nM rapamycin. At the end of the exposure period, cells were stained with annexin V–APC and propidium iodide (PI), according to the manufacturer’s instruction (BioLegend). Flow cytometry was carried out on a BD LSRII Fortessa (BD Biosciences) with optimised parameters (Supplementary Fig. 1). Data were collected using BD FACSDiva, version 8.0, and analysed with FlowJo X, version 10.0.7r2 (FlowJo).

### Human Pericyte Contraction and Calcium Imaging During Chemical Ischemia

We utilised chemical ischemia (hereinafter referred to as ischemia) with antimycin A and sodium iodoacetate to block oxidative phosphorylation and glycolysis, to study the effect of 30 nM of rapamycin on human pericyte contraction and Ca^2+^ flux during ischemia. We chose this model as we could add pharmacological inhibitors of energy-producing cellular pathways to mimic energy depletion that occurs in ischemia [2], without the need for modulating oxygen in our live imaging setup. For all live single-cell imaging experiments, primary HBVPs were cultured on glass coverslips and imaged on a Nikon Ti Live Cell Microscope and imaged using the same methodology as described previously [21], with modification. Experiments with HBVP were performed between passages 6 and 8. HBVPs were incubated with Fura 2-AM ratiometric calcium indicator (1 μM, Life Technologies) diluted in imaging buffer (1X HBSS) (no Ca^2+^, no Mg^2+^, no phenol red), 15 mM HEPES, 30 mM glucose, 1 mM MgCl_2_, 2 mM CaCl_2_ in Milli-Q H2O, pH 7.4) and maintained at 37 °C for 10 min. HBVPs were imaged using differential interference contrast (DIC) microscopy on a temperature-controlled 37 °C upright Nikon Ti Live Cell Microscope with a × 40 oil immersion objective and EMCCD camera (Photometrics Evolve 512 × 512). Pericytes were only imaged provided there were no other interfering cells in the field of view. For contractility assessment, following baseline images, imaging buffer was removed and replaced with imaging buffer containing vehicle (0.1% v/v DMSO), chemical ischemia solution (5 μM antimycin A, Sigma #A8674; 500 μM sodium iodoacetate, Sigma; concentrations determined from [23]), and rapamycin (30 nM, Adooq Bioscience). Our rat experimental data had shown that 10 nM and 100 nM of rapamycin were equally effective in reducing the rate of pericyte contraction to OGD. Therefore, prior to commencing human pericyte experiments, we tested several rapamycin concentrations within this range and chose 30 nM as the optimal concentration after preliminary experiments. Live DIC images (to visualise cell area) and fluorescent (340-nm and 380-nm excitation and dual 510-nm emission; to visualise Ca^2+^ flux) images were recorded at 1-min intervals. Seven to 12 cells were imaged per coverslip within the same session with an automated stage facilitating repeated return to their *XY* coordinates for recording over time. Images were processed with NIS Elements Analysis software and exported to ImageJ for further analysis.

To determine the extent of change relative to baseline over a period of time, cell area and Ca^2+^ flux were normalised to baseline to generate normalised cell area (*A*/*A*_0_) and normalised Ca^2+^ flux (*R*/*R*_0_), as described in previous literature [21]. To describe the directionality and relative magnitude of this response over time, net area under the curve (nAUC) was calculated in GraphPad Prism using the formula ΔX*([(Y1+Y2)/2]−baseline] where baseline is defined as *Y* = 0, defining both peaks above and below the baseline as peaks. The difference was computed by subtracting the area of peaks below the baseline from the area of peaks above the baseline.

### Middle Cerebral Artery Occlusion (MCAO) and Cerebral Blood Flow Measurement

All animal procedures were approved by the University of Tasmania Animal Ethics Committee (A0016160 and A0018608) and were compliant with the Australian NHMRC Code of Practice for the Care and Use of Animals for Scientific Purposes. Transient intraluminal filament MCAO was performed for 60 min followed by 30 min of reperfusion in adult 3–4-month-old male NG2-DsRed mice as previously described [24]. Transient MCAO was chosen for this study as the restoration of blood flow is needed to assess whether capillaries can receive blood and if blood flow is restricted at pericyte locations, which is not possible in the permanent model. At the commencement of MCAO, mice were randomised and treated with an IP injection of either 1 mg/kg rapamycin or saline (vehicle control) (full methodological details in the Supplementary Materials).

### Tissue Fixation and Processing

After 30 min of reperfusion, mice were terminally anaesthetised via IP injection of pentobarbital (300 mg/kg) and transcardially perfused at 9.6 mL/min with 37 °C PBS + heparin (1 unit/mL; DBL Heparin Sodium Injection BP, Pfizer) for 1 min and then PBS + 4% w/v paraformaldehyde (Sigma) for 1 min, followed by PBS + 0.1% w/v FITC-albumin (Sigma) + 5% w/v gelatin (Sigma) for 1 min to obtain a microvascular cast of perfused vessels, as described previously [25]. Mice were covered in ice for 30 min to set the gelatin, after which brains were removed, incubated in PBS + 4% PFA for 1 h at 4 °C, washed 3× in PBS, and cryoprotected in PBS + 30% w/v sucrose (Sigma) for 24 h at 4 °C. Brains were rapidly frozen by placing them in moulds with Epredia Cryomatrix embedding resin (Thermo Fisher) on a floating foam tube holder on liquid nitrogen and then stored at − 80 °C. Brains were sectioned coronally at 40 μm with a CM1850 cryostat (Leica Biosystems) at − 18 °C, and tissue slices were placed directly onto slides for analysis.

### Blood Vessel Labelling Immunohistochemistry

Slide-mounted tissue was permeabilised with 0.3% v/v Triton X-100 (Sigma) in Dako Antibody Diluent (Agilent) for 40 min at room temperature. Tissue was blocked using Dako Serum-Free Protein Block (Agilent) for 60 min at room temperature. Isolectin GS-IB4-Alexa Fluor 647 conjugate (Thermo Fisher) was diluted 1:500 in Dako Antibody Diluent and incubated with the tissue for 24 h at 4 °C in a humidified dark chamber. Sections were then washed 3 × 10 min in PBS, before being incubated in 1X TrueBlack (Biotium) for 1 min to reduce the impact of autofluorescence; washed 3 × 2 min in PBS; rinsed in distilled water; and then coverslipped with ProLong Gold antifade reagent with DAPI (Thermo Fisher).

### Image Acquisition and Analysis

Slides were imaged using a VS120 slide scanner (Olympus, Japan) as described previously [25]. After an overview (DAPI, Ex 388 nm; Em 448 nm; × 2 magnification), tracing of tissue sections and focus-map generation, slides were scanned using extended focus imaging (15-μm total range, 3-μm spacing) at × 20 magnification in the DAPI (Ex 388 nm; Em 488 nm), FITC (Ex 494 nm; Em 530 nm), Texas red (Ex 576 nm; Em 625 nm), and Cy5 (Ex 650 nm; Em 670 nm) channels. Optimum exposure times were initially determined manually and then kept consistent for all images. Images were acquired from coronal sections equivalent to Bregma + 0 mm. A total of four 500 µm^2^ ROIs were placed, one in the mediolateral striatum and one in the primary somatosensory cortex of each hemisphere.

Images were processed in ImageJ and analysed blinded to treatment group using independently developed ImageJ macro scripts to allow semi-automated analysis and reduce image analysis bias (https://github.com/brownls23/NG2DsRed-IHC-analysis-.git). To detect NG2-DsRed-positive capillary pericytes, binarised DAPI and NG2-DsRed soma-specific overlays were generated to calculate pericyte number, with a manual binary threshold matching step, DAPI-positive cell selection step, and removal of smooth muscle cell NG2DsRed signal step. FITC-albumin-specific overlays were generated with a manual binary threshold matching step and removal of large vessels. These images were used to analyse perfused vessel width underneath pericyte soma, with vascular association confirmed by positive ILB4 labelling. Width was defined as the minimum vascular width of FITC-albumin labelling beneath a NG2-DsRed-positive pericyte soma and measured using the Straight-Line tool in ImageJ. Where one pericyte spanned two vessel branches, width of both vessels was measured. Where there was no FITC-albumin signal present or FITC-albumin signal stopped under a pericyte soma, perfused vessel width under pericyte was given a value of 0 μm. For open/closed analysis of perfused vessel width, width measurements were binned into two groups: 0 μm and > 0 μm. *N* = 1086 individual vessel width measurements from *N* = 10 animals were taken across both treatment groups (ipsilateral striatum: *N* = 1–18, contralateral striatum: *N* = 24–50, ipsilateral cortex: *N* = 10–49, contralateral cortex: *N* = 25–50, vessels per animal).

### Statistical Analysis

Data were processed in Microsoft Excel, and all statistical analysis was performed using GraphPad Prism 10 (GraphPad, USA). When data was tested for outliers, a ROUT test (*Q* = 1%) was used and outliers were removed from data sets unless they were biologically relevant. When comparing at least three independent variables, data were tested for normality of the residuals using the D’Agostino and Pearson test or the Shapiro–Wilk test if *n* numbers were too small. When this data was normally distributed, variables were compared using ordinary one-way ANOVA with Dunnett’s multiple comparisons test between groups. When this data was not normally distributed, variables were compared using the Kruskal-Wallis test with Dunn’s multiple comparisons test between groups. When comparing how a response is affected by two factors, normality and sphericity were assumed, and variables were compared using two-way ANOVA with either Dunnett’s or Sidak’s multiple comparisons test between groups. When comparing how a response is affected by two factors where one of the factors was repeated, independence, normality, and sphericity were assumed, and variables were compared using repeated measures (RM) two-way ANOVA with Sidak’s multiple comparisons test between groups. When comparing frequency of two categorical variables, contingency table matrices were used and variables compared using Fisher’s exact test. A *p* < 0.05 was considered statistically significant. Statistical tests used for each analysis are reported in figure legends. For ANOVAs, overall ANOVA results are reported in figure legends and results of post hoc tests are reported in the ‘Results’ section and are represented in each figure.

## Results

### Rapamycin Reduced mTORC1 Activity in Rat Pericytes During Normoxia and Oxygen Glucose Deprivation (OGD)

Cultured pericytes expressed their typical markers PDGFRβ and desmin (Supplementary Fig. 2). We confirmed successful mTORC1 inhibition with rapamycin treatment by measuring the phosphorylation of the downstream target of mTOR, S6 ribosomal protein (Supplementary Fig. 3). Full membrane scans of blots can be found in Supplementary Figs. 4–9.

### Rapamycin Reduced Rat Pericyte Contractility Without Affecting Pericyte Cell Death

**Fig. 1:**
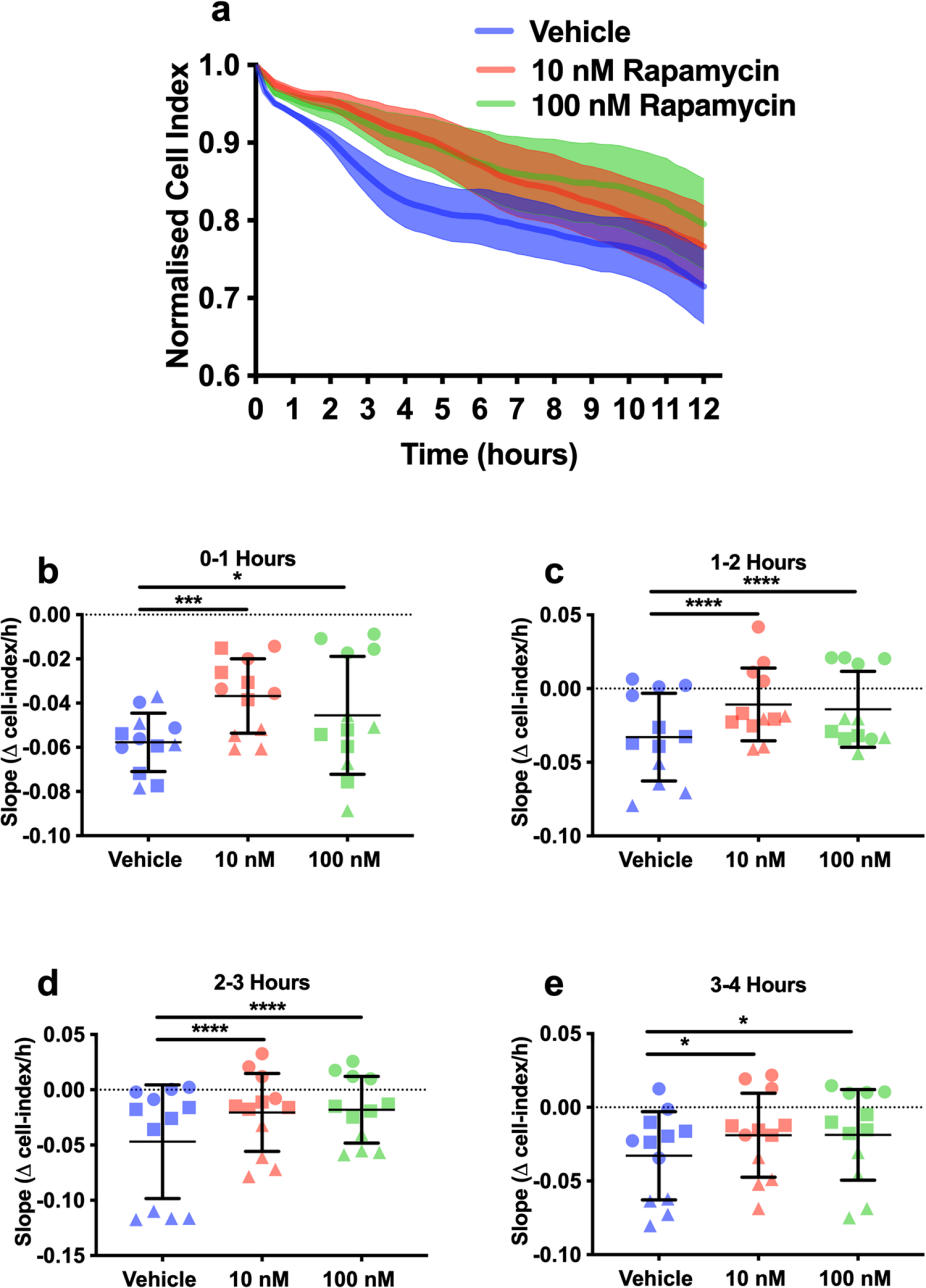
Rapamycin reduces pericyte contractility during OGD. **A** Average normalised cell index for vehicle (blue), 10 nM rapamycin– (red) and 100 nM rapamycin– (green) treated cells during 12 h of OGD. **B** Slope of cell index 0–1 h post-OGD, vehicle (blue), 10 nM rapamycin (red), and 100 nM rapamycin (green); circle, square, and triangles signify 3 independent cultures, 4 wells per culture. A two-way ANOVA was conducted with culture and treatment as variables. Treatment, *F* (2, 27) = 9.716, *p* = 00007; culture, *F* (2, 27) = 19.04, *p* < 0.0001; interaction, *F* (4, 27) = 7.275, *p* = 0.0004, with Tukey’s multiple comparisons tests to assess the main treatment effects. *p < 0.05, ***p <0.001. **C** Slope 1–2 h post-OGD. Two-way ANOVA, treatment, *F* (2, 27) = 20.38, *p* < 00001; culture, *F* (2, 27) = 119.3, *p* < 0.0001; interaction, *F* (4, 27) = 3.985, *p* = 0.0114, with Tukey’s multiple comparisons tests to assess the main treatment effects. ****p < 0.0001. **D** Slope 2–3 h post-OGD. Two-way ANOVA, treatment, *F* (2, 27) = 28.13, *p* < 00001; culture, *F* (2, 27) = 209.5, *p* < 0.0001; interaction, *F* (4, 27) = 9.048, *p* < 0.0001, with Tukey’s multiple comparisons tests to assess the main treatment effects. ****p < 0.0001. **E** Slope 3–4 h post-OGD. Two-way ANOVA, treatment, *F* (2, 27) = 4.526, *p* = 0.0202; culture, *F* (2, 27) = 68.8, *p* < 0.0001; interaction, *F* (4, 27) = 0.7043, *p* = 0.5959, with Tukey’s multiple comparisons tests to assess the main treatment effects. *p < 0.05

We analysed the effects of ischemia and rapamycin treatment on pericyte contraction using an electrical impedance system (iCELLigence) [23]. Exposure of rat pericytes to 12 h of OGD resulted in a decline in cell index, which indicated cell contraction (Fig. 1A). This contraction was due to the ischemia rather than the media change, as the pericyte cell index did not decline after changing media to normoxia media (Supplementary Fig. 10). We tested the effects of rapamycin treatment on the slope of the cell index curve (indicating the rate of pericyte contraction). Both doses of rapamycin significantly reduced the slope of the cell index curve between 0–1, 1–2, 2–3, and 3–4 h of OGD (0–1 h: Fig. 1B; 10 nM: − 0.04 ± 0.02, 100 nM: − 0.05 ± 0.03 versus vehicle: − 0.06 ± 0.01, 10 nM: *p* = 0.0004, 100 nM: *p* = 0.04; 1–2 h: Fig. 1C; 10 nM: − 0.01 ± 0.03, 100 nM: − 0.01 ± 0.03 versus vehicle: − 0.03 ± 0.03, 10 nM: *p* < 0.0001, 100 nM *p* < 0.0001; 2–3 h: Fig. 1D; 10 nM: − 0.02 ± 0.04, 100 nM: − 0.02 ± 0.03 versus vehicle: − 0.05 ± 0.06, 10 nM: *p* < 0.0001, 100 nM: *p* < 0.0001; 3–4 h: Fig. 1E; 10 nM: − 0.02 ± 0.03, 100 nM: − 0.02 ± 0.03 versus vehicle: − 0.03 ± 0.03, 10 nM: *p* = 0.04, 100 nM: *p* = 0.04).

**Fig. 2:**
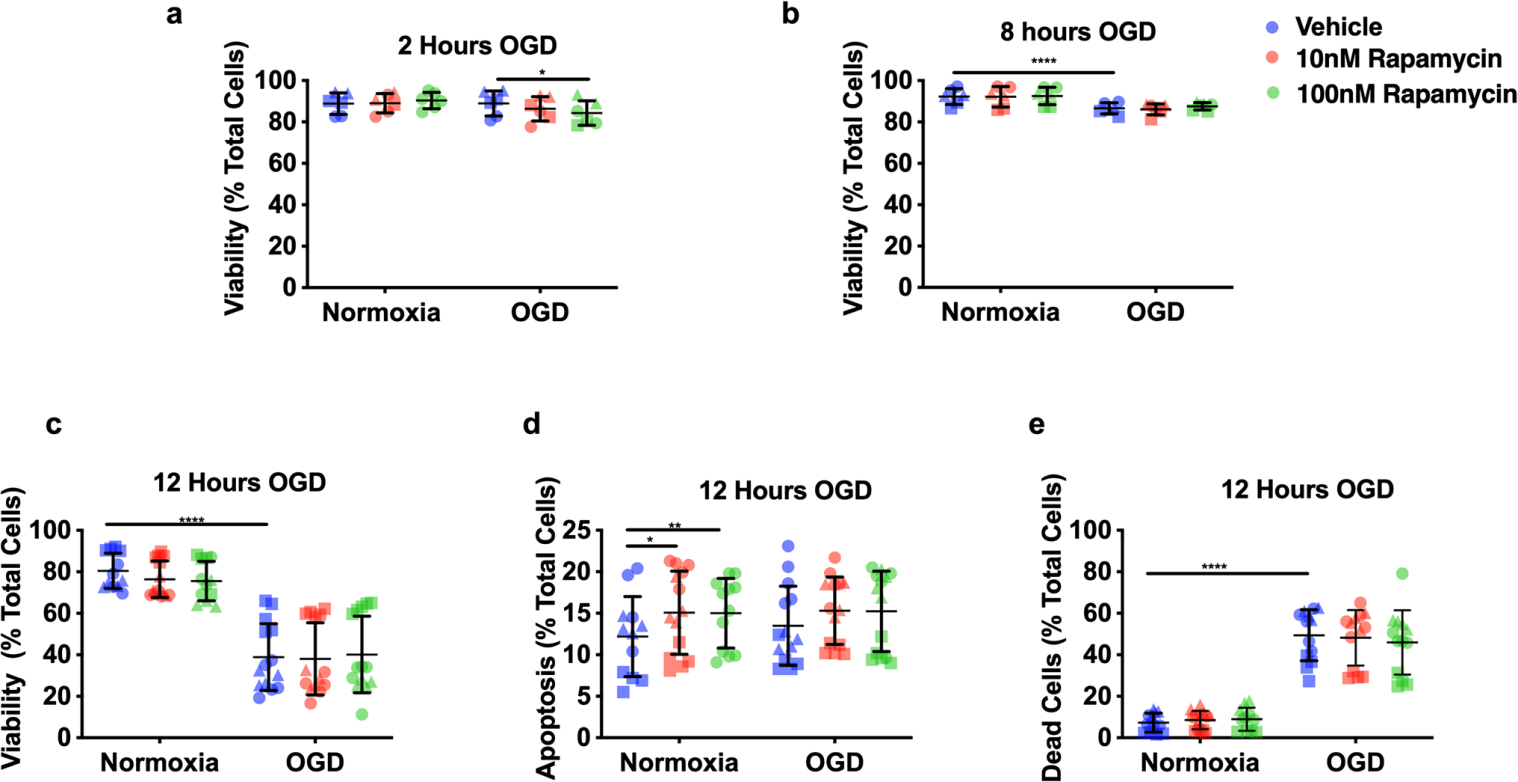
Rapamycin did not affect pericyte cell death during OGD. Vehicle (blue), 10 nM rapamycin (red), and 100 nM rapamycin (green); circle, square, and triangles signify 3 independent cultures, 2–5 wells per culture. **A** Pericyte viability after 2-h OGD (% of cells negative for annexin V (AV) and propidium iodide (PI) staining). Two-way ANOVA, treatment, *F* (5, 18) = 5.717, *p* = 0.0025; culture, *F* (2, 18) = 69.72, *p* < 0.0001; interaction, *F* (10, 18) = 0.7912, *p* = 0.6382, with Sidak’s multiple comparisons tests to assess the main treatment effects. *p < 0.05. **B** Pericyte viability after 8-h OGD. Two-way ANOVA, treatment, *F* (5, 18) = 49.59, *p* < 0.0001; culture, *F* (2, 18) = 125.8, *p* < 0.0001; interaction, *F* (10, 18) = 5.105, *p* = 0.0014, with Sidak’s multiple comparisons tests to assess the main treatment effects. *p < 0.0001. **C** Pericyte viability after 12-h OGD. Two-way ANOVA, treatment, *F* (10, 62) = 280.5, *p* < 0.0001; culture, *F* (2, 62) = 257, *p* < 0.0001; interaction, *F* (10, 62) = 6.682, *p* < 0.0001, with Sidak’s multiple comparisons tests to assess the main treatment effects. ****p < 0.0001. **D** Apoptosis of pericytes after 12-h OGD (% cells positive for AV but negative for PI). Two-way ANOVA, treatment, *F* (5, 62) = 4.604, *p* = 0.0012; culture, *F* (2, 62) = 113.9, *p* < 0.0001; interaction, *F* (10, 62) = 2.797, *p* = 0.0064, with Sidak’s multiple comparisons tests to assess the main treatment effects. *p < 0.05, **p < 0.01. **E** Pericyte cell death 12 h after OGD (% cells positive for PI +/− annexin V). Two-way ANOVA, treatment, *F* (5, 68) = 215.1, *p* < 0.0001; culture, *F* (2, 58) = 90.85, *p* < 0.0001; interaction, *F* (10, 58) = 5, *p* < 0.0001, with Sidak’s multiple comparisons tests to assess the main treatment effects. ****p < 0.01

We performed flow cytometry for propidium iodide and annexin V (see Supplementary Fig. 1 for example heat maps), to confirm that the observed effect of rapamycin was due to its effect on contraction, rather than a consequence of changes in cell death and/or detachment. There was no reduction in cell viability at 2 h of OGD (Fig. 2A; OGD vehicle: 88.9 ± 6.0% versus normoxia vehicle: 88.9 ± 5.2%, *p* > 0.99), suggesting that the reduction in cell index reflects pericyte contraction rather than cell death and the subsequent detachment. Ten nanomolar rapamycin had no effect on pericyte viability, while 100 nM rapamycin caused a small but statistically significant reduction in viability at 2 h in the OGD group (Fig. 2A; 10 nM: 86.4 ± 5.9%, 100 nM: 84.3 ± 5.9% versus vehicle: 88.9 ± 6.0%, 10 nM: *p* = 0.3, 100 nM *p* = 0.01). No significant pericyte death was recorded until 8 and 12 h of OGD (Fig. 2B–E; 12-h OGD vehicle: 38.9 ± 16.1% versus normoxia vehicle: 80.4 ± 8.5%, *p* < 0.001). Rapamycin had no effect on pericyte viability in the normoxia or OGD group at 12 h (Fig. 2C; OGD,10 nM: 38.9 ± 17.5%, 100 nM: 40.2 ± 18.7% versus vehicle: 38.9 ± 16.1%, 10 nM: *p* = 0.99 100 nM *p* = 0.98). Rapamycin caused a small but statistically significant increase in pericyte apoptosis in the normoxia group at 12 h (Fig. 2D; normoxia 10 nM: 15.1 ± 5.4%, 100 nM: 15 ± 4.6% versus vehicle: 12.2 ± 4.8%, 10 nM: *p* = 0.007, 100 nM *p* = 0.01). Rapamycin had no effect on pericyte death in the normoxia or OGD group at 12 h (Fig. 2E; OGD 10 nM: 47.5 ± 15.3%, 100 nM: 45.3 ± 15.6% versus vehicle: 38.9 ± 16.1%, 10 nM: *p* = 0.94, 100 nM *p* = 0.3).

### Rapamycin Prevented Human Pericyte Contractility to Ischemia but Did Not Inhibit Calcium Flux

**Fig. 3:**
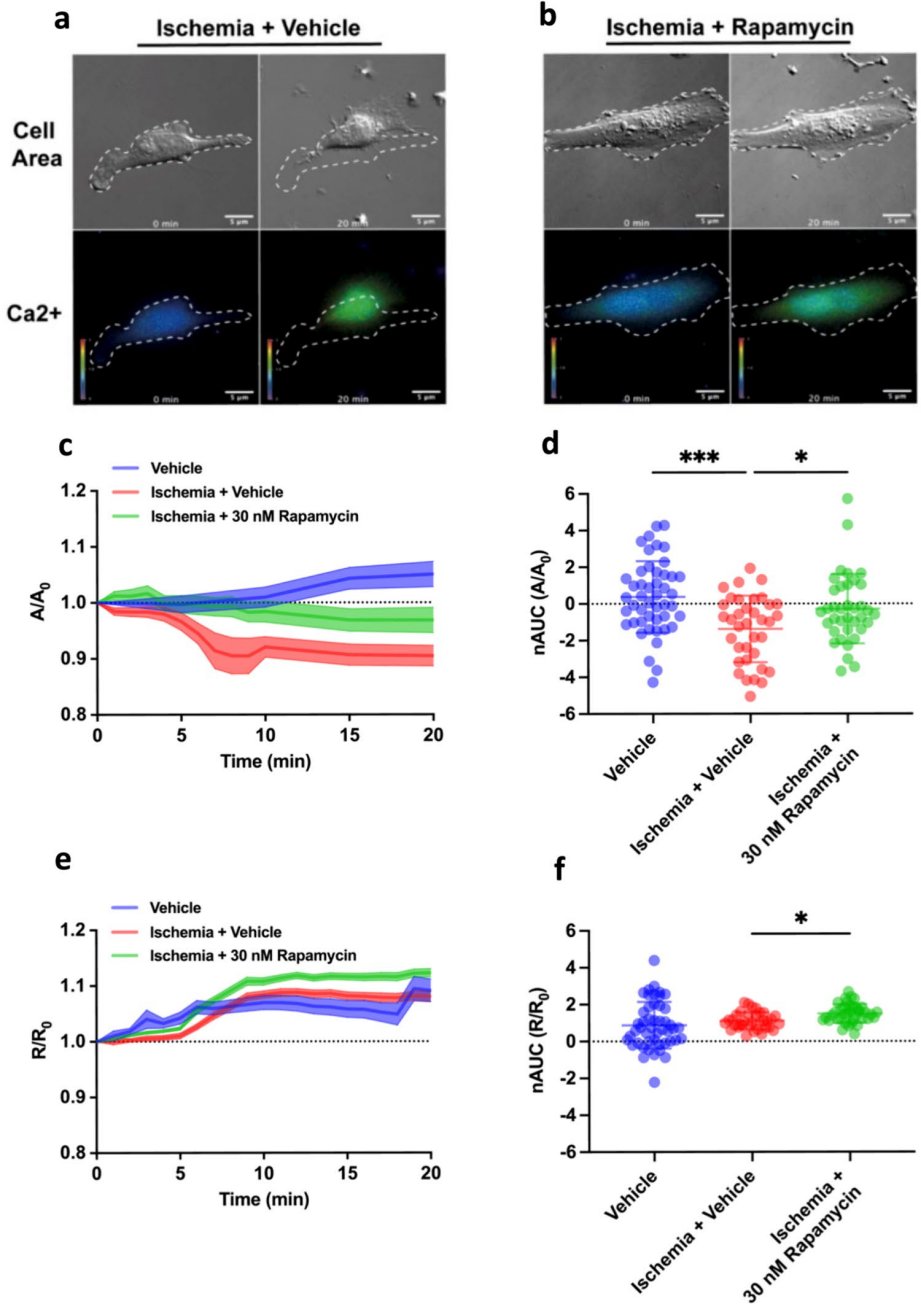
Rapamycin prevents pericyte contraction in response to chemical ischemia. Representative DIC and fluorescent Fura-2 AM Ca^2+^ flux images of pericytes treated with **a** chemical ischemia + vehicle (DMSO) or **b** rapamycin, at baseline (0 min) and 20-min time points. The white dotted outline indicates cell membrane boundaries for each representative cell at baseline. Colorimetric scale represents intracellular Ca^2+^ levels with the ratiometric Fura-2 AM Ca^2+^ indicator. Lower right scale bar = 10 μm. For each cell: **c** normalised cell area (*A*/*A*_0_) and **e** normalised Fura-2 AM calcium flux (*R*/*R*_0_), compared to baseline, were calculated over time following each treatment. The dotted line represents baseline cell area or calcium flux. The dark lines represent the mean and the shaded area represents the SEM for each treatment group. Number of individual cells analysed for each group: vehicle (DMSO) (*N* = 47); ischemia (*N* = 36); ischemia + Rap (rapamycin) (*N* = 37); over 6 replicate cultures. **d** Net area under the curve (nAUC) of *A*/*A*_0_ and **f** *R*/*R*_0_ was calculated for each treatment group. Bars represent mean ± SD. One-way ANOVA (treatment, *F* (2,117) = 8.785, *p* = 0.0003) with Dunnett’s multiple comparisons test was used to compare *A*/*A*_0_ groups. The Kruskal-Wallis test (treatment, *H* (2) = 15.28, *p* = 0.0005) with Dunn’s multiple comparisons test was used to compare *R*/*R*_0_ groups. *p < 0.05, ***p < 0.001

We wanted to determine if calcium levels were associated with pericyte contraction in response to ischemia, as suggested earlier [2, 23], and whether rapamycin could modulate this effect. For these experiments, we used human brain vascular pericytes (HBVPs) since they were used in our previous work [23]. We tested the effects of ischemia and rapamycin treatment on the net area under the curve (nAUC), an indicator of the extent of pericyte cell membrane retraction (contraction), in a single-cell imaging assay we have used previously [21]. Exposure of human pericytes to ischemia resulted in a retraction of the cell membrane after 20 min of ischemia (Fig. 3a, c) and a corresponding significant reduction in normalised nAUC over time compared to control treatment (Fig. 3d; ischemia + vehicle: − 1.4 ± 1.8 versus vehicle: 0.4 ± 1.9, *p* < 0.001). Rapamycin significantly reduced pericyte membrane contraction (Fig. 3b, c) and significantly increased nAUC compared to ischemia alone (Fig. 3d; ischemia + rapamycin: − 0.3 ± 1.9 versus ischemia + vehicle: − 1.4 ± 1.8, *p* = 0.03). We then assessed whether rapamycin affected Ca^2+^ changes in pericytes in response to ischemia using the calcium indicator Fura-2. Ischemia did not change in Ca^2+^ flux compared to vehicle alone (ischemia: 1.0 ± 0.6 versus vehicle: 0.89 ± 1.3, *p* = 0.8). Rapamycin caused a small but significant increase in calcium flux during ischemia compared to ischemia alone (Fig. 3e, f; rapamycin + ischemia: 1.5 ± 0.5 versus ischemia: 1.0 ± 0.6, *p* = 0.01). These results suggest that rapamycin can prevent ischemia-induced pericyte contraction downstream of calcium entry into the cells.

### Rapamycin Increases Vessel Width and the Number of Open Vessels in the Striatum After MCAO

**Fig. 4:**
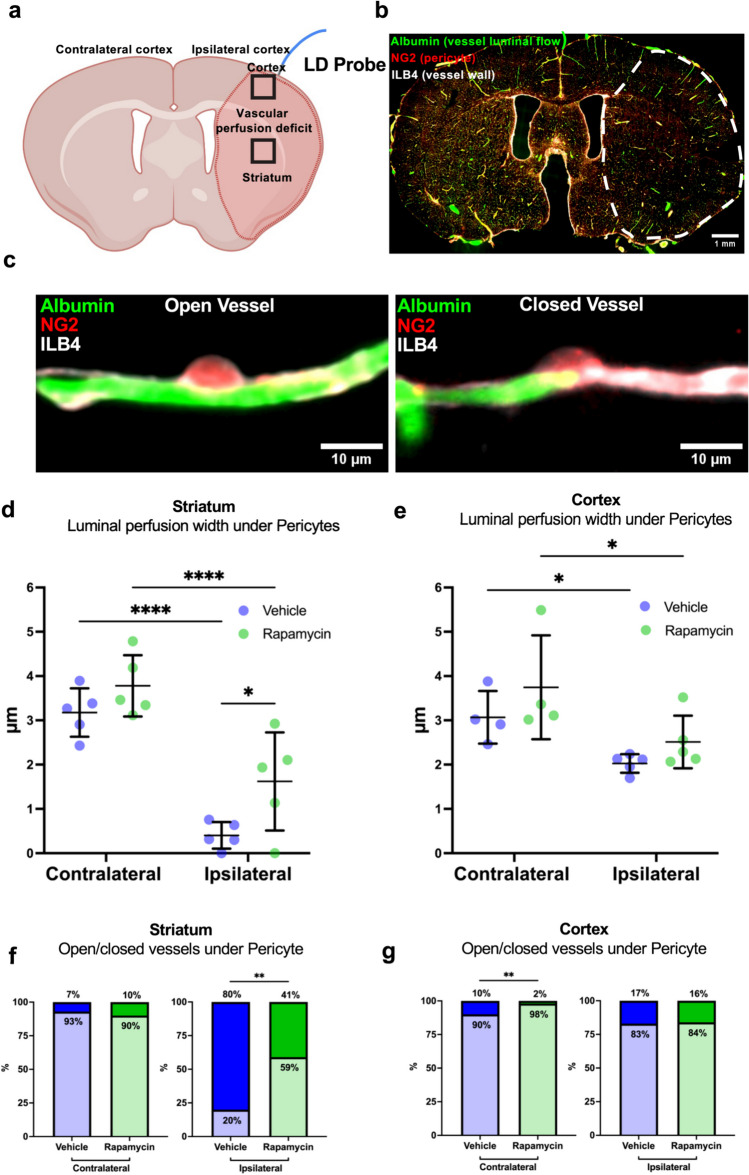
Rapamycin increases luminal width and the proportion of open vessels in the striatum following MCAO. **a** Schematic of mouse brain coronal section under bregma demonstrating contralateral and ipsilateral hemispheres, relative location of laser doppler (LD) probe, the area of vascular perfusion deficit in the ipsilateral hemisphere, and regions of interest sampled. **b** Example fluorescent image of whole coronal brain section labelled with FITC-albumin (green), NG2DsRed (Red), and isolectin B4 (ILB4, white), with the area of vascular perfusion deficit annotated with white dotted line. **c** Example images of capillaries with luminal FITC-albumin labelling (left) and reduced luminal FITC-albumin labelling (right) under pericyte soma. **d**, **e** Quantification of mean perfused vessel luminal width under pericytes in the striatum and cortex in both the contralateral and ipsilateral hemispheres following 60-min MCAO and 30-min reperfusion. Individual data points are presented as mean ± SD and were determined from 1086 individual vessel width measurements. Striatum (ipsilateral *N* = 1–18, contralateral *N* = 24–-50) and cortex (ipsilateral *N* = 10–49, contralateral *N* = 25–50) vessels per animal. Repeated measures two-way ANOVA (**d** hemisphere, *F* (1,8) = 162.9, *p* < 0.0001; treatment, *F* (1,8) = 4.831, *p* = 0.0592, and **e** hemisphere, *F* (1,5) = 38.93, *p* < 0.0015; treatment, *F* (1,9) = 2.961, *p* = 0.1194) with Sidak’s multiple comparisons test was used to compare treatment and hemisphere groups. When values were missing, a mixed-effects model with Sidak’s multiple comparisons test was used. **f**, **g** Vessel data (from **d**, **e**) were binned based on luminal width being 0 (closed) or > 0 (open) in the striatum and cortex. Data are presented as a percentage of open or closed vessels. Fisher’s exact test was used to compare groups. *p < 0.05, **p < 0.01, ****p < 0.0001

Given that rapamycin can delay ischemia-induced contraction in pericytes (Figs. 1 and 3), we next investigated whether rapamycin could affect vessel width under pericytes following MCAO. Laser Doppler flowmetry monitoring of blood flow in the upper layers of the somatosensory cortex did not reveal any change in blood flow during reperfusion with rapamycin treatment (Supplementary Fig. 11). However, laser Doppler flowmetry can only measure overall flow coming from all vessels in a small volume of tissue directly below the probe. We therefore performed a microvascular luminal cast using FITC-albumin perfusion to analyse changes in individual vessel perfused luminal width beneath pericyte soma after treatment with rapamycin [25]. The area of perfusion deficit during ischemia in this model encompasses the MCA territory which includes the striatum and the cerebral cortex containing the somatosensory cortex; these regions were chosen for analysis (Fig. 4a). Despite 30 min of recanalisation of the MCA following 60-min MCAO, there appeared to be areas devoid of FITC-albumin luminal labelling, suggesting that some capillaries were closed (Fig. 4b). Interestingly, many of these areas with closed vessels (vessels devoid of luminal labelling) appeared to be associated with pericytes (Fig. 4c).

Analysis of luminal width underneath pericyte soma revealed that MCAO had caused a substantial decrease in vessel width in the ipsilateral striatum compared to the corresponding contralateral hemisphere (Fig. 4d; ipsilateral vehicle: 0.4 ± 0.3 µm versus contralateral vehicle: 3.2 ± 0.5 µm, *p* < 0.0001). Rapamycin significantly increased luminal width in the ipsilateral striatum compared to vehicle (Fig. 4d; rapamycin: 1.6 ± 1.1µm versus vehicle: 0.4 ± 0.3µm, *p* = 0.03). Rapamycin did not affect luminal width in the contralateral striatum compared to vehicle (Fig. 4d; rapamycin: 3.8 ± 0.7 µm versus vehicle: 3.2 ± 0.5 µm, *p* = 0.4). There was a significant decrease in vessel width in the ipsilateral cortex compared to the corresponding contralateral hemisphere (Fig. 4e, ipsilateral vehicle: 2.0 ± 0.2 µm versus contralateral vehicle: 3.1 ± 0.6 µm, *p* = 0.0111). However, rapamycin did not significantly alter vessel width compared to vehicle in the ipsilateral (rapamycin: 2.5 ± 0.6 µm versus vehicle: 2.0 ± 0.2 µm, *p* = 0.2) or contralateral (rapamycin: 3.7 ± 1.2 µm versus vehicle: 3.1 ± 0.6 µm, *p* = 0.3) hemispheres (Fig. 4e).

It was notable that some vessels had no FITC-albumin signal indicative of closed vessels both in the striatum and cortex following MCAO. We therefore quantified the proportion of open (presence of some FITC-albumin signal under pericytes) and closed (zero FITC-albumin signal under pericytes) vessels. Consistent with the vessel width data, the ipsilateral striatum only had 20% of capillaries open while the contralateral striatum had 90% of capillaries open. Rapamycin significantly increased percentage of open capillaries compared to vehicle (Fig. 4f; rapamycin: 59% versus vehicle: 20%, *p* = 0.0059). Rapamycin did not significantly alter the percentage of open capillaries in the ipsilateral cortex (Fig. 4g; rapamycin: 84% versus vehicle: 83%, *p* = 0.9). Interestingly, rapamycin significantly increased the percentage of open capillaries in the contralateral cortex compared to vehicle (Fig. 4g; rapamycin: 98% versus vehicle: 90%, *p* = 0.002).

### MCAO Induces Pericyte Loss Which Is Not Prevented by Rapamycin

**Fig. 5:**
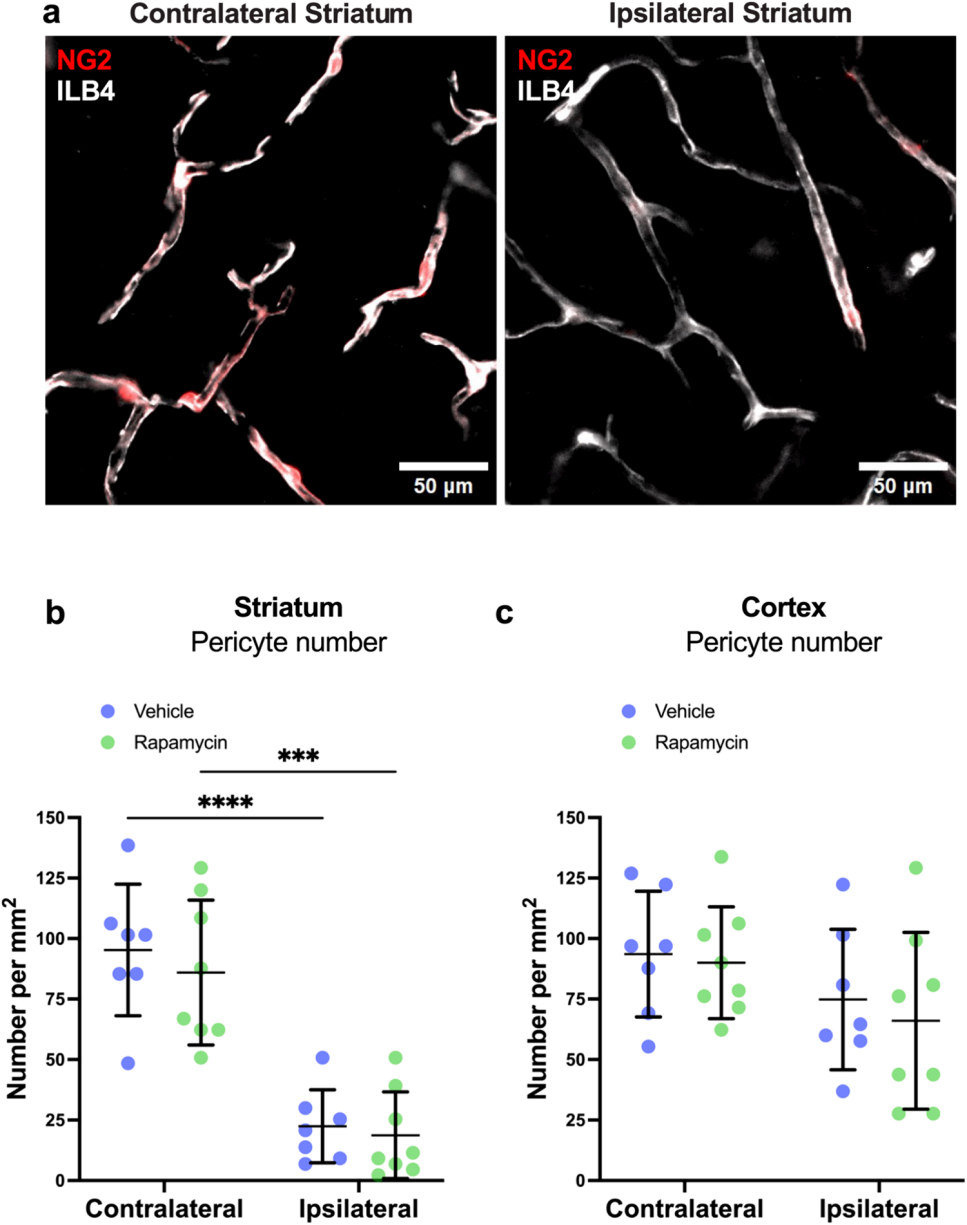
Rapamycin had no effect on the reduction in pericyte number in the striatum following MCAO. **a** Example images of capillaries (ILB4, white) with pericytes (NG2, red) in contralateral (left) and ipsilateral (right) striatum of mouse subject to 60-min MCAO followed by 30-min reperfusion. **b**, **c** Quantification of pericyte number in the striatum and cortex. Individual data points represent individual mice overlayed with mean ± SD. Vehicle treatment *N* = 7, rapamycin treatment *N* = 8. Two-way ANOVA (**b** hemisphere, *F* (1,13) = 70.43, *p* < 0.0001; treatment, *F* (1,8) = 0.5430, *p* = 0.4743, and **c** hemisphere, *F* (1,13) = 4.579, *p* = 0.0519; treatment, *F* (1,13) = 0.2980, *p* = 0.5944) with Tukey’s multiple comparisons test was used to compare groups. ***p<0.001, ****p < 0.0001

Our in vitro data had shown that rapamycin does not reduce pericyte death in response to OGD (Fig. 2), and so we determined whether rapamycin could prevent pericyte loss following MCAO. We found that pericyte number, as defined by the presence of NG2DsRed signal on vessels, was reduced in the ipsilateral compared to the contralateral striatum (Fig. 5a, b; ipsilateral vehicle: 22 ± 15 pericytes/mm^2^ versus contralateral vehicle: 95 ± 27 pericytes/mm^2^, *p* < 0.0001). Rapamycin did not significantly affect pericyte number in the striatum of either the ipsilateral (rapamycin: 19 ± 18 pericytes/mm^2^ versus vehicle: 22 ± 15 pericytes/mm^2^, *p* = 0.99) or contralateral (rapamycin: 86 ± 30 pericytes/mm^2^ versus vehicle: 95 ± 27 pericytes/mm^2^, *p* = 0.8) hemispheres (Fig. 5b). There was no significant change in pericyte number in the ipsilateral cortex compared to the contralateral cortex (Fig. 5c; ipsilateral vehicle: 75 ± 29 pericytes/mm^2^ versus contralateral vehicle: 94 ± 26 pericytes/mm^2^, *p* = 0.6). Rapamycin had no significant effect on pericyte numbers in the cortex of either the ipsilateral (rapamycin: 66 ± 37 pericytes/mm^2^ versus vehicle: 75 ± 29 pericytes/mm^2^, *p* = 0.9) or contralateral (rapamycin: 90 ± 23 pericytes/mm^2^ versus vehicle: 94 ± 26 pericytes/mm^2^, *p* = 0.99) hemispheres (Fig. 5c).

## Discussion

This is one of few reports of a potential therapeutic intervention aimed at reducing pericyte contraction in response to ischemia, and it is the first study to report that rapamycin can reduce pericyte contraction. Rapamycin reduced cultured pericyte contraction during ischemia in vitro using both OGD and ischemia paradigms. We demonstrate that rapamycin exerts its anti-contractile effect downstream of intracellular calcium changes. Furthermore, these effects were independent of changes in pericyte cell death. Finally, we highlight the translational potential of rapamycin by showing that it increases capillary diameter at pericyte locations and the number of open capillaries in mouse brains following MCAO.

Our results, along with previous in vitro investigations, indicate that pericytes contract before they die. We demonstrated that pericytes begin to contract within an hour of OGD onset but do not begin to die until at least 7 h later. These results are in line with our laboratory’s previously published work, which showed that HBVPs exposed to ischemia begin to contract within an hour after treatment onset but do not die until 24 h later [23]. Hall *et al*. [2] showed that pericytes in cortical slices begin to contract 15 min after the onset of ischemia and die in significant numbers after 1 h. Hall *et al.* [2] reported that pericytes die at 24 h after 90 min of MCAO, but the timeframe of pericyte contraction was not measured in their study. Meanwhile, Yemisci *et al.* [5] reported pericyte constriction of capillaries at 6 h of reperfusion following 2-h MCAO but did not report pericyte cell death in vivo. Here, we showed that MCAO significantly reduced capillary diameter at pericyte locations and decreased the number of open vessels in the ipsilateral striatum 30 min following recanalisation. These findings are broadly consistent with those of Yemisci *et al.* [5]. Furthermore, a recent study by Qiu et al. using two-photon imaging found that there was significant vasoconstriction in the areas of vessels covered by pericyte somas as well as processes at 3 h post-recanalisation [26]. Staehr et al [27] reported that 1 h of cortical photothrombosis stroke led to constriction of pericytes in the peri-infarct cortex at 3 and 24 h after stroke which was associated with a lack of perfusion is these capillaries. The capillaries that still had flow showed an increase prevalence of capillary stalling (stalled red blood cells in a capillary). Similarly, Shrouder et al. [4] showed that 87% of pericytes constrict during 60 min of intraluminal MCAO ischemia and remain constricted following reperfusion in mice. This constriction was significantly associated with capillary stalls at pericyte soma during ischemia and out to 24 h of reperfusion. These findings demonstrate that pericytes present a promising therapeutic target to counteract no-reflow in stroke.

Rapamycin treatment significantly slowed pericyte contraction during OGD and increased capillary diameter at pericyte soma and increased the number of open capillaries at pericyte soma following MCAO. This strongly suggests that rapamycin is reducing both the contraction of pericytes in vitro and pericyte constriction of vessels in vivo. This is the first study to report that rapamycin can reduce pericyte contraction/constriction. One previous study managed to influence capillary diameter by using nanoparticles that contained adenosine conjugated to the lipid squalene (which allowed a prolonged circulation of this nucleoside) in mice that were subjected to MCAO/reperfusion. Among other observed neuroprotective effects, the adenosine nanoparticles could prevent capillary stalling, which could suggest potential therapeutic effects; however, the role of pericytes in adenosine effects was not specifically investigated [28].

Rapamycin’s anti-contractile effect does not appear to be mediated by changes in pericyte cell death in vitro as pericytes did not die until approximately 8–12 h of OGD, and rapamycin had no effect on cell death at any time point investigated. This is in line with previous studies showing that cultured brain pericytes undergo rapid cell cycle arrest [29], but do not die during 4 h of OGD [29, 30]. Previous studies investigating other cell types showed that rapamycin can reduce cell death in cultured neurons [16] and cultured brain endothelial cells [17] during OGD. Furthermore, rapamycin has been shown to reduce astrocyte proliferation, migration, and production of inflammatory mediators to OGD in vitro [18]; reduce blood-brain barrier permeability; and reduce infarct volume in animal models of stroke [13, 15]. The lack of pericyte protection in vitro afforded by rapamycin in this study may be explained by the hypothesis that OGD itself down-regulates mTORC1 in vehicle-treated cells, which means that by the time the pericytes start to die between 8 and 12 h, there would be no additional mTORC1 inhibition effected by rapamycin treatment.

Although we did not directly assess pericyte cell death in vivo, we did observe a significant reduction in NG2DsRed signal following 60 min of MCAO and 30 min of reperfusion. This could be suggestive of early pericyte cell injury leading to pericyte membrane shedding or leakage of the cytoplasm through damaged membranes. Shrouder et al. [4] also reported a loss of EGFP signal from pericytes at 90 min following reperfusion; however, they did not directly investigate pericyte cell death using TUNEL staining until 24 h, which revealed an increase in pericyte death at this time point. Therefore, it remains to be determined if pericyte cell death occurs as early as 30 and 90 min following reperfusion. We did, however, show there was no significant difference in the reduction of NG2DsRed signal between vehicle and rapamycin treatment groups. This could suggest that rapamycin has little influence on pericyte damage in the early phases of reperfusion following stroke*.*

We showed that rapamycin reduced the contraction of human pericytes in response to ischemia without inhibiting influx of intracellular calcium. Previous studies showed that pericyte contraction during ischemia in brain slices was dependent on rapid extracellular calcium entry through voltage-gated calcium channels (VGCCs) [2] and that derivatives of rapamycin can reduce the opening of VGCCs in hippocampal neurons [31], while rapamycin itself did not exert activity at VGCCs [32]. In this study, we found only a small increase in calcium levels between ischemia alone and ischemia plus rapamycin at the time of maximal effect of rapamycin on contraction. This suggests that rapamycin could be influencing pericyte contractility downstream of calcium entry/release. One potential mechanism is that rapamycin is reducing the sensitivity of the contractile apparatus of the pericytes to calcium by modulating signalling pathways such as the Rho-A/ROCK pathway that regulate myosin-light-chain phosphorylation [33]. Shrouder et al. [4] demonstrated that topical application of the ROCK inhibitor fasudil over the stroke cortex could reverse constriction of capillaries by pericytes and capillary stalls during 70-min MCAO and at 90 min and 24 h of reperfusion in mice. This is in line with our findings that pericyte constriction can be reversed and highlights the important role of the Rho-A/ROCK pathway in inducing pericyte constriction of capillaries post-stroke. Rho-A has been shown to regulate the contractility of bovine retinal pericytes via myosin light chain kinase and alpha-smooth muscle actin [34] and to induce brain pericyte contraction in cortical slices and in vivo [35, 36]. Rapamycin has been shown to reduce Rho-A expression and activation via mTORC1 inhibition and subsequent reduction in S6 kinase and eukaryotic translation initiation factor 4E-binding protein 1 (4EBP1) activity to reduce cytoskeletal rearrangement in human rhabdomyosarcoma and Ewing sarcoma cell lines [33]. Therefore, future studies will investigate the role of Rho-A/ROCK in rapamycin’s ant-icontractile effects on pericytes in stroke.

In conclusion, we found that rapamycin reduces the rate of pericyte contraction during OGD and ischemia, and the reduction in contraction was mediated through a mechanism that is independent of changes in intracellular calcium. Complementing our in vitro findings, we found that rapamycin also increased the diameter of capillaries at pericyte soma and increased the number of open capillaries in the striatum post-recanalisation in mice. This strongly suggests that rapamycin also reduces pericyte constriction of capillaries following stroke. These findings have important therapeutic relevance for preventing no-reflow after successful reopening of occluded arteries after ischemic stroke.

## Supplementary Information

Below is the link to the electronic supplementary material.

Supplementary file1 (DOCX 29.5 KB)

## Data Availability

The data that support the findings of this study are available from the authors upon reasonable request.
