# Supplementary material for: Rapamycin Treatment Reduces Brain Pericyte Constriction in Ischemic Stroke
Source: Transl Stroke Res. 2024 Sep 27;16(4):1185–97. doi: 10.1007/s12975-024-01298-x (PMC12202568; doi:10.1007/s12975-024-01298-x)
Supplement: Supplementary file 1 — Supplementary file1 (DOCX 29.5 KB) [file 12975_2024_1298_MOESM1_ESM.docx]

**Rapamycin Treatment Reduces Brain Pericyte Constriction in Ischemic Stroke**

Translational Stroke Research

Daniel J Beard^1,2#*^, Lachlan S Brown^3#^, Gary P Morris^3^, Yvonne Couch^1^, Bryan A Adriaanse^4^, Christina Simoglou Karali^5^, Anna M Schneider^1^, David W Howells^3^, Zoran B Redzic^6^, Brad A Sutherland^3#*^, Alastair M Buchan^1#^

^1^ Acute Stroke Programme, Radcliffe Department of Medicine, University of Oxford, Oxford, UK

^2^ School of Biomedical Sciences and Pharmacy, University of Newcastle, Newcastle, Australia

^3^ Tasmanian School of Medicine, College of Health and Medicine, University of Tasmania, Hobart, Australia

^4^ Nuffield Department of Clinical Neurosciences, University of Oxford, UK

^5^ Department of Oncology, University of Oxford, UK

^6^ Department of Physiology, Faculty of Medicine, Kuwait University, Kuwait

* Corresponding authors:

Dr Daniel Beard

Email: [daniel.j.beard@newcastle.edu.au](mailto:daniel.j.beard@newcastle.edu.au)

Email: [brad.sutherland@utas.edu.au](mailto:brad.sutherland@utas.edu.au)

# denotes co-first and co-senior authorship

Supplementary Information

**Supplementary methods**

***Cell Culture***

Briefly, each pericyte culture was produced from 5 individual rat brains from two- to three-month old Wistar rats. After cell extraction from the brain, pericytes were cultured under standard incubation conditions (37^°^C; 5% CO2 in 95% humidified air) as described earlier [1], but without addition of Puromycin. Thus, both BECs and pericytes initially grew in cell culture medium that consisted of DMEM, 20% FBS, 1% penicillin/streptomycin, 0.1mg/mL ascorbic acid for 10 days with a full media change on Day 1, followed by a half media change every second day (P0 culture). Ten days after plating, pericytes largely overgrew BECs, at which point cells were passaged to uncoated, poly-L-lysine (Sigma Aldrich) treated 6 well plates and allowed to grow to ~70% confluence before being used for experimental procedures (P1 culture). This step eliminated BECs from cultures, as they could not produce enough basal lamina components and thus, do not attach to uncoated plastic [2].

***Immunocytochemistry***

Rat pericytes were seeded at 30,000 cells per well in 8 well Millicell® EZ Slides (Millipore) and allowed to adhere. After fixation (4% paraformaldehyde for 10 min), autofluorescence quenching (1% sodium borohydride for 10 min) and blocking (10% donkey serum for 1 h), the cultured cells were incubated overnight at 4^°^C in 1% serum/PBS that contained the following primary antibodies: cluster of differentiation 31 (CD31; goat polyclonal; R&D Systems; 1:100), beta-type platelet-derived growth factor receptor (PDGFRβ; goat polyclonal; R&D Systems; 1:100) and desmin (rabbit polyclonal; AbCam; 1:100). After washing, the cultures were incubated for 2 h at room temperature in the dark with secondary antibodies (anti-goat and anti-rabbit Alexa488-conjugated; AbCam; 1:400). Cytoskeletal actin was counterstained using Alexa594-conjugated phalloidin (Sigma-Aldrich; 1:500) and nuclei were counterstained with DAPI (Sigma-Aldrich). Images were acquired using a Zeiss Axioscope 2 (Carl Zeiss) with appropriate filter settings and acquisition parameters.

***Western Blotting***

Cells were lysed in cell lysis buffer through cell scraping, incubated at 4°C for 30 min and centrifuged at 4°C for 30 min at 11,700 g. Supernatant was collected for Western blotting. Protein concentration (mg/mL) of cell lysates were determined (RD DC Protein Assay, Biorad using a DC (Biorad, UK). 10 μg of sample per well was run on NuPAGE 4-12% Bis-Tris gels (Invitrogen) and then transferred to nitrocellulose using an iBlot2 dry transfer system (Invitrogen). Membranes were blocked in 5% BSA for 1 h at room temperature. Membranes were immunoblotted with primary antibodies against phospho-S6 at Ser235/236 (p-S6; rabbit monoclonal; Cell Signaling Technology; 1:2000), total S6 protein (t-S6; rabbit monoclonal; Cell Signalling Technology; 1:8000), and α-Tubulin (mouse monoclonal; Abcam; 1:4000) diluted in 5% BSA 0.1% TX-100 and incubated overnight at 4^o^C. Secondary antibodies (HRP-conjugated, goat anti-rabbit or goat anti-mouse immunoglobulin, 1:1000, Dako) were applied to the membranes for 1 h at room temperature. The immunoblots were visualised and quantified on a Biorad ChemiDoc^TM^ MP imaging system (Biorad) using ECL-advanced detection reagents (Invitrogen). The density of the bands was measured using Biorad Image Lab software v6.0.1 (Biorad). The loading controls were performed by analysis of the t-S6 protein and α-tubulin protein. S6 phosphorylation at Ser235/236 (an index of S6 kinase activity and readout of upstream mTORC1 activity) was expressed as the ratio of S6 phosphorylation to t-S6 protein, to account for variability in t-S6 protein between samples.

***Middle Cerebral Artery Occlusion and Cerebral Blood Flow Measurement***

Adult 3–4-month-old male NG2-DsRed mice (Jackson Laboratories #008241) were used for all MCAO experiments. All animal procedures were approved by the University of Tasmania Animal Ethics Committee (A0016160 and A0018608) and were compliant with the Australian NHMRC Code of Practice for the Care and Use of Animals for Scientific Purposes. Mice were housed in standard conditions with ad libitum access to food and water and on a 12 h light:dark cycle (light phase was 7:00 AM-7:00 PM). The temporary intraluminal filament MCAO model (Longa method) was performed as previously described [3] followed by 30 minutes of reperfusion. Mice were anaesthetised with isoflurane at 5% in O2 delivered at 1 L/min in a box for induction, and 2-4% in O_2_ delivered at 0.5 L/min intranasally through a nose cone for maintenance (Advanced Anaesthesia Specialists, Australia). A silicone-coated filament (602145PK10; 20-25 g mouse, 602345PK10; 25-35 g mouse; Doccol Corporation, USA) was advanced into the right external carotid artery and up the internal carotid artery to occlude the origin of the right MCA. At the commencement of MCAO, mice were treated with an IP injection of either 1 mg/kg rapamycin (Sapphire Bioscience #A10782-10MM-D) or saline (vehicle control). After 60 min, the filament was retracted to allow recanalisation of the MCA and reperfusion of the brain. To measure cerebral blood flow (CBF) during the MCAO and reperfusion periods, a laser Doppler probe (moorVMS-LDF1, Moor Instruments) was positioned in a silicon probe holder (PHDO, Moor Instruments) perpendicular to the right temporal skull at 3 mm lateral and 1.5 mm caudal to bregma. Occlusion was confirmed by a sudden drop in CBF to <30% of baseline.

***Blinding, animal numbers, and exclusions***

Animals were randomly assigned to treatment and surgical groups (rapamycin vs vehicle (saline); MCAO vs sham) and researchers were blinded to treatment groups by preparing treatments in identical vials and assigning treatments to arbitrary groups A and B. Animals were excluded if a blood flow drop to <30% of baseline during MCAO was not exhibited, they were not able to achieve a stable anesthetic plane, or a terminal bleed or haemorrhage occurred as a result of the surgery. Out of 28 animals that were entered into the study, there were 4 exclusions.

***Laser Doppler signal processing and analysis***

LD data were recorded at 100Hz using LabScribe (IWorks) and down sampled to 0.1Hz for export (bins of 10 second data) for each animal to determine changes in CBF during common carotid artery occlusion (CCAO, middle cerebral artery occlusion (MCAO), middle cerebral artery reperfusion (MCAR), and common carotid artery reperfusion (CCAR). Baseline traces were cut to a flat 5-minute baseline section prior to CCAO and normalised to the mean over that baseline period. All CCAO periods were normalised to the last 5 minutes prior to MCAO. All MCAR periods were normalised to the first 2 minutes following MCAR.

**Supplementary Figure**s*
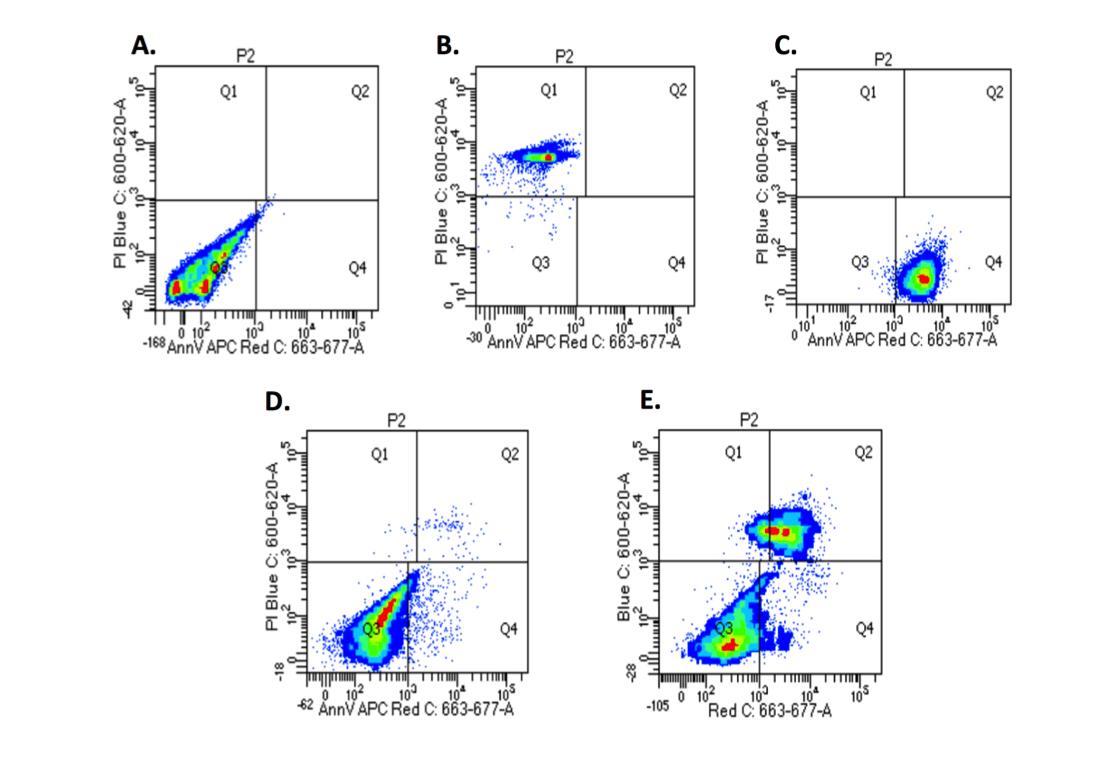
*

**Supplementary Fig 1** Flow cytometry heat maps. A. Unstained control cells. B. Propodium Iodide (PI) control cells. C. Annexin V (AV) control cells. D. Vehicle Normoxia fully stained cells. E. Vehicle Oxygen Glucose Deprivation (OGD) fully stained cells. Note the increase in the number of cells in quadrant 2 (Q2) indicative of PI-positive and AV-positive cells.

**
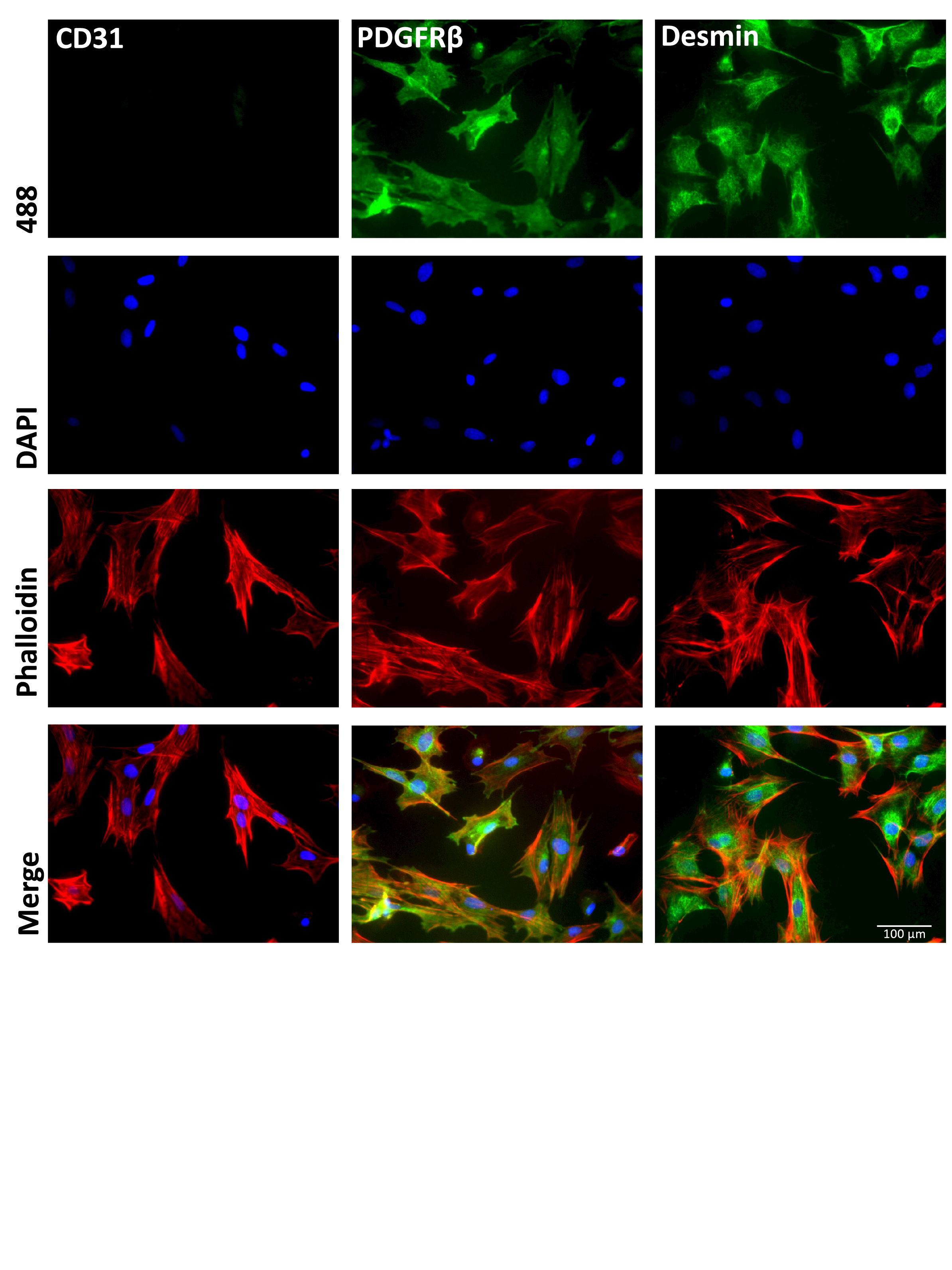
**

**Supplementary Fig 2** Immunocytochemistry of primary rat brain pericytes. Pericytes were fixed in 4% paraformaldehyde and stained for endothelial (CD31), and pericyte (PDGFRβ and Desmin) markers (all green – Alexa488) to confirm a pericyte phenotype. Cells were counterstained with a cytoskeletal marker (phalloidin – red) and a nuclear marker (DAPI – blue).

**
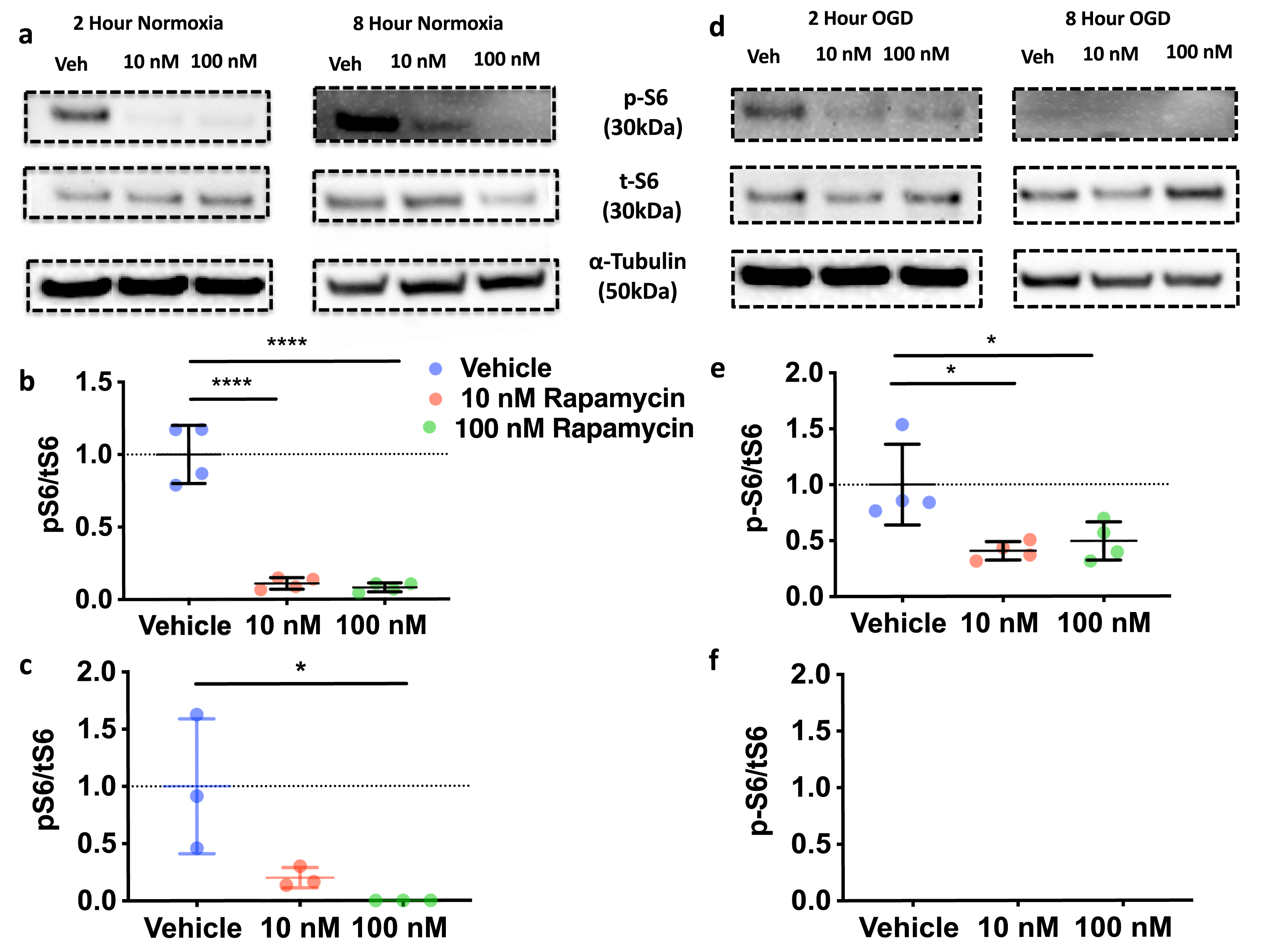
**

**Supplementary Fig 3** Effect of rapamycin treatment on mTORC1 activity. (**a**) Representative blots for pS6, t-S6 and loading control, tubulin after 2 h and 8 h of Normoxia. (**b**) Quantification of p-S6 relative to t-S6; (indicating mTOR activity) after 2 h normoxia. One way ANOVA (Treatment, F(2,9) = 76.26, p < 0.0001) with Dunnett’s test multiple comparisons test to compare treatment groups. (**c**) Quantification of p-S6 relative to t-S6 after 8 h normoxia. Kruskal Wallis: H (2) = 7.448, p = 0.0036 with Dunn’s multiple comparisons test to compare treatment groups. (**d**) Representative blots after 2 h and 8 h of OGD. (**e**) Quantification of p-S6 relative to t-S6 after 2 h OGD. Kruskal Wallis (Treatment, H (2) = 7.538, p = 0.0107) with Dunn’s multiple comparisons test to compare treatment groups. (**f**) Quantification of p-S6 relative to t-S6 after 8 h OGD. Each value was mean ± SD from 3-4 samples (split over 3-4 gels/blots). * p < 0.05, **** p < 0.0001.


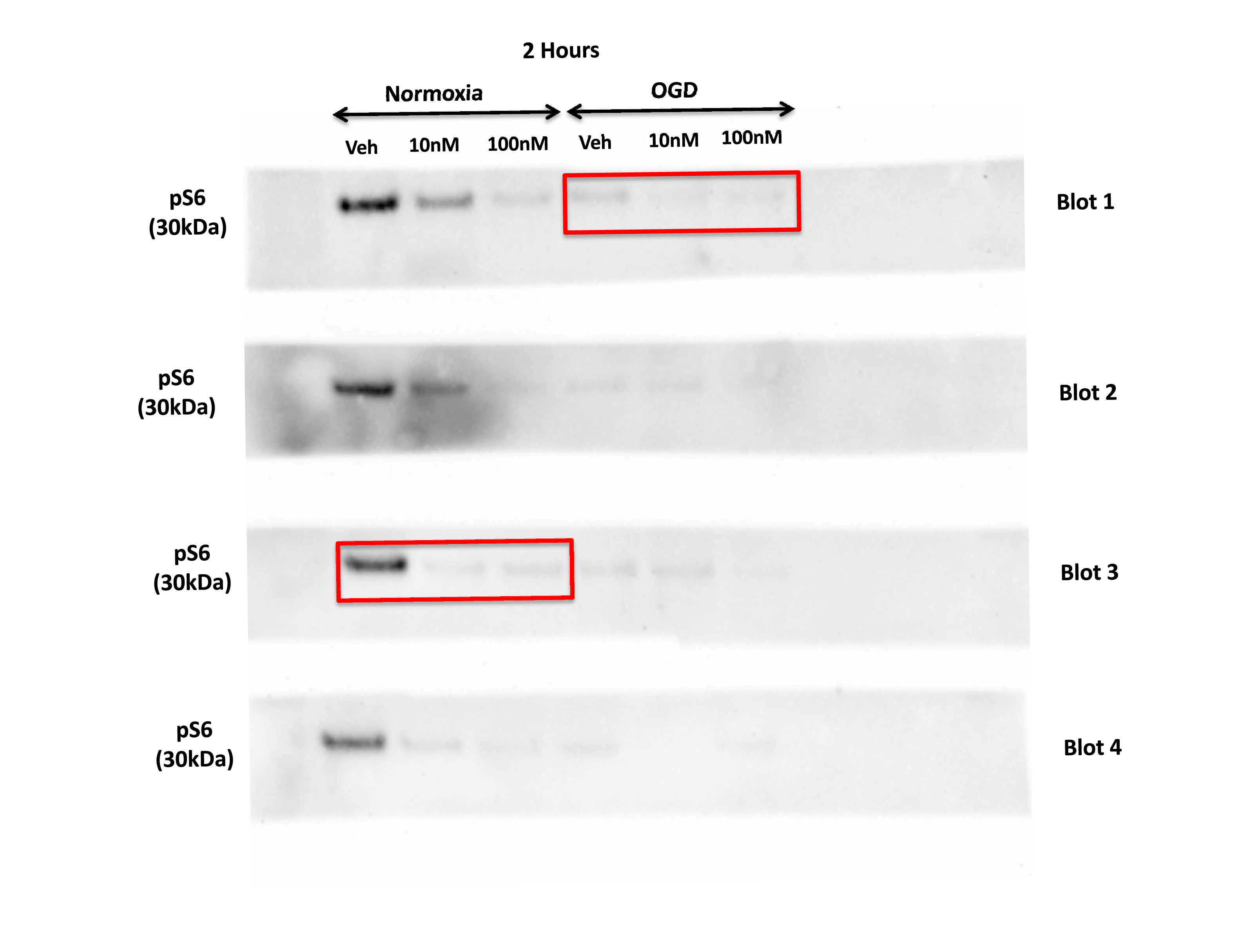


**Supplementary Fig 4** Full unedited blots for Supplementary Fig 3 a,d– used for the analysis of Phospho-S6 (pS6) in pericytes exposed to 2 hours of normoxia or Oxygen Glucose Deprivation (OGD). The panels are chemiluminescent images taken using a Biorad ChemiDoc^TM^ MP imaging system, which provides information of the molecular weight/size of the bands (weights depicted to left of blots). The red boxes indicate the bands featured in Fig. 2a,d. The signals of the bands from the original, unprocessed immunoblots were measured using using Biorad Image Lab software v6.0.1. Note, the brightness and contrast of the representative blots in Fig 2 a,d has been enhanced for visualisation purposes only.

*
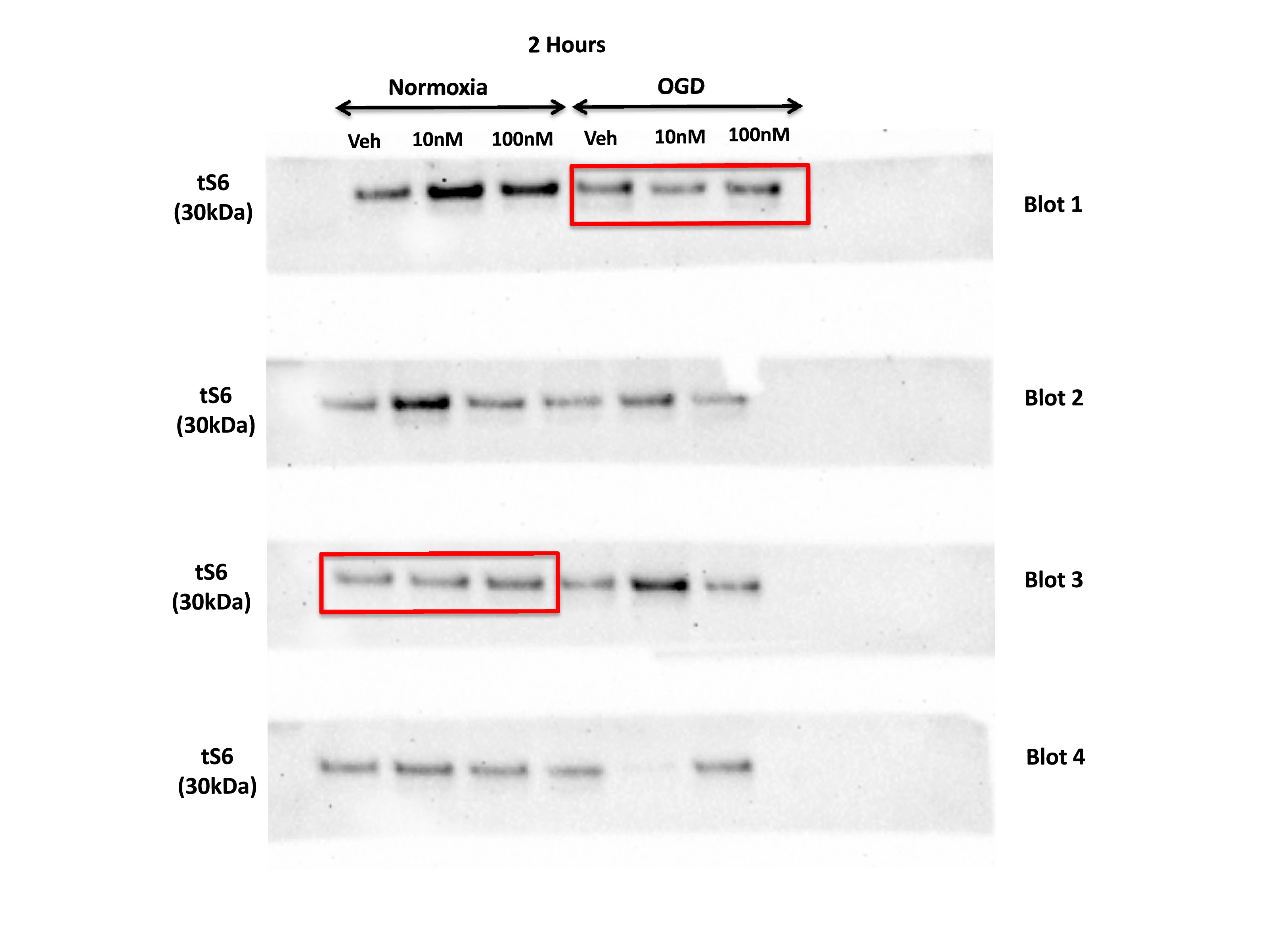
*

**Supplementary Fig 5** Full unedited blots for Supplementary Fig 3 a,d– used for the analysis of Total-S6 (tS6) in pericytes exposed to 2 hours of normoxia or Oxygen Glucose Deprivation (OGD). The panels are chemiluminescent images taken using a Biorad ChemiDoc^TM^ MP imaging system, which provides information of the molecular weight/size of the bands (weights depicted to left of blots). The red boxes indicate the bands featured in Fig 2 a,d. The signals of the bands from the original, unprocessed immunoblots were measured using using Biorad Image Lab software v6.0.1. Note, the brightness and contrast of the representative blots in Fig 2 a,d has been enhanced for visualisation purposes only.

*
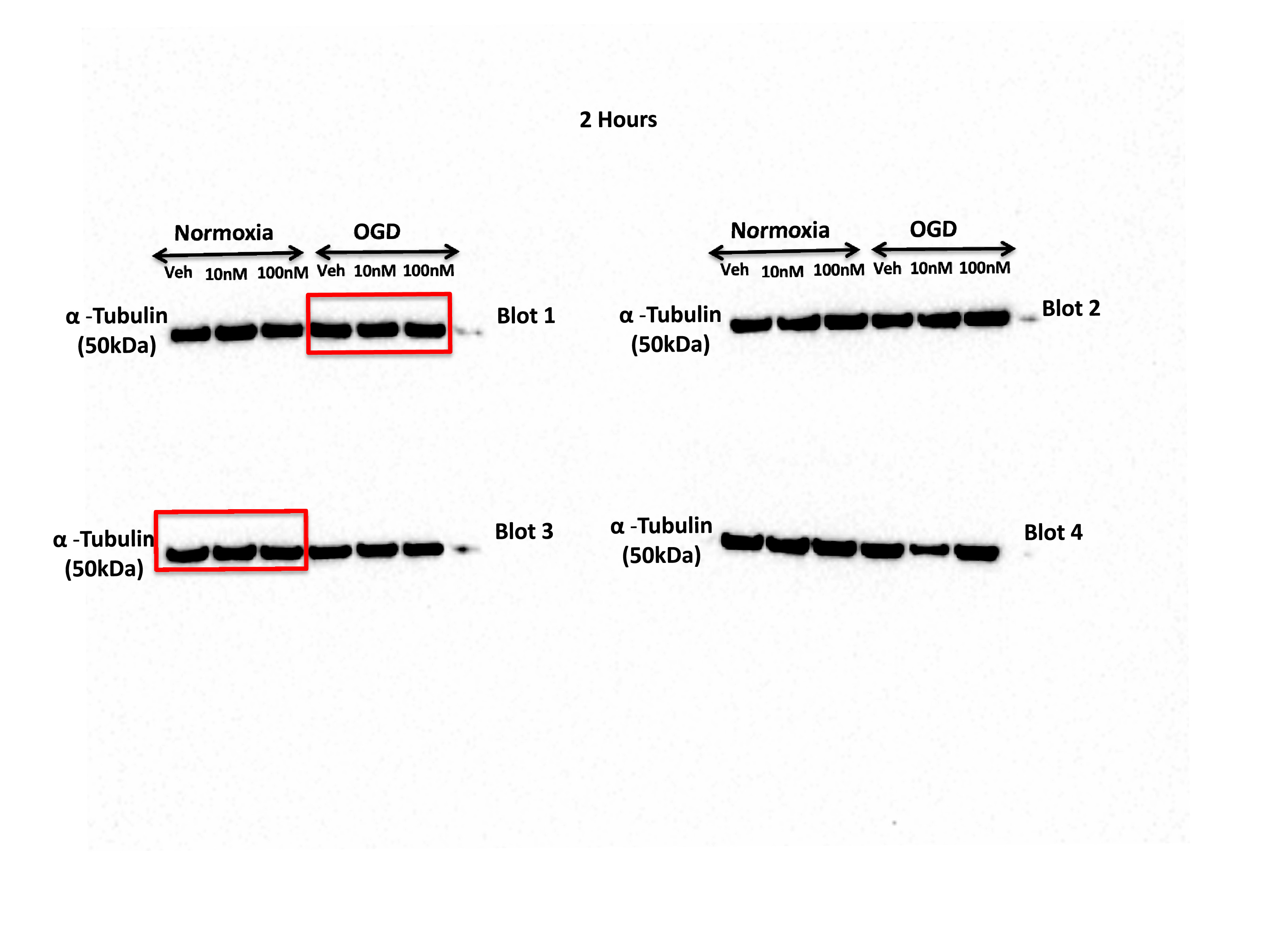
*

**Supplementary Fig 6** Full unedited blots for Supplementary Fig 3 a,d– used for the analysis of alpha-tubulin in pericytes exposed to 2 hours of normoxia or Oxygen Glucose Deprivation (OGD). The panels are chemiluminescent images taken using a Biorad ChemiDoc^TM^ MP imaging system, which provides information of the molecular weight/size of the bands (weights depicted to left of blots). The red boxes indicate the bands featured in Fig 2 a,d. The signals of the bands from the original, unprocessed immunoblots were measured using using Biorad Image Lab software v6.0.1. Note, the brightness and contrast of the representative blots in Fig 2 a,d has been enhanced for visualisation purposes only.

*
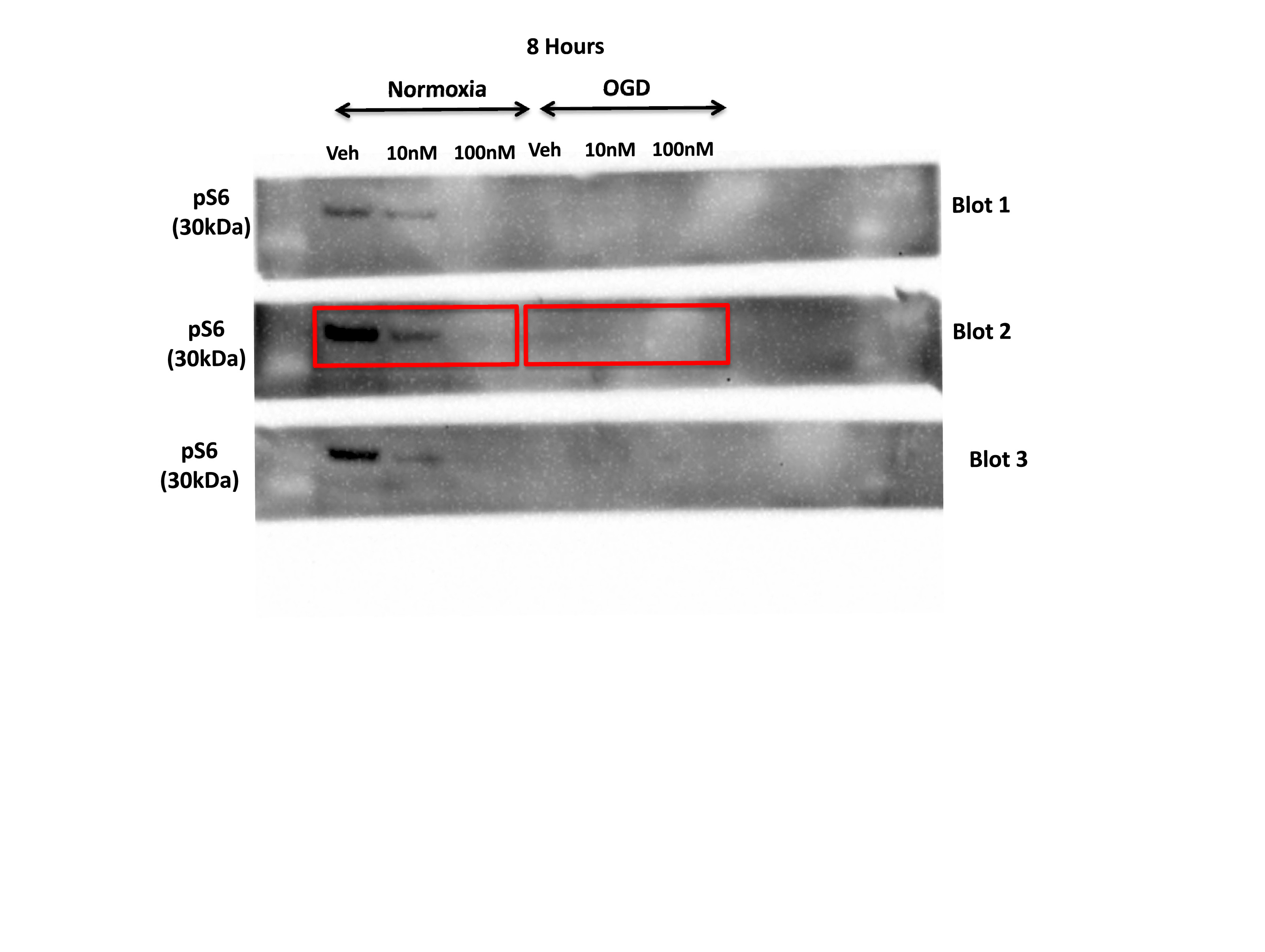
*

**Supplementary Fig 7** Full unedited blots for Supplementary Fig 3 a,d– used for the analysis of Phospho-S6 (pS6) in pericytes exposed to 8 hours of normoxia or Oxygen Glucose Deprivation (OGD). The panels are chemiluminescent images taken using a Biorad ChemiDoc^TM^ MP imaging system, which provides information of the molecular weight/size of the bands (weights depicted to left of blots). The red boxes indicate the bands featured in Fig. 2 a,d. The signals of the bands from the original, unprocessed immunoblots were measured using using Biorad Image Lab software v6.0.1. Note, the brightness and contrast of the representative blots in Fig 2 a,d has been enhanced for visualisation purposes only.

*
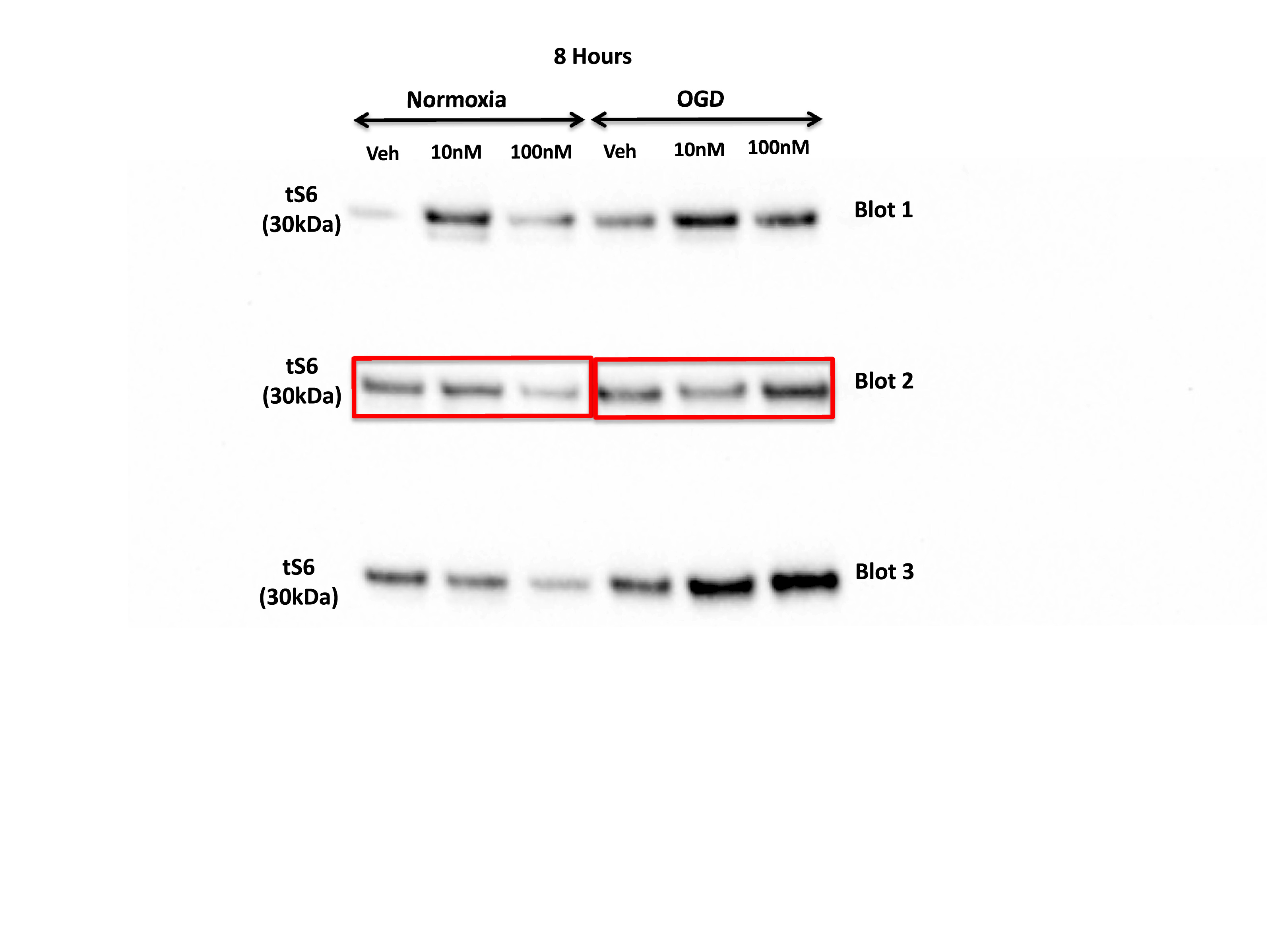
*

**Supplementary Fig 8** Full unedited blots for Supplementary Fig 3 a,d– used for the analysis of Total-S6 (tS6) in pericytes exposed to 8 hours of normoxia or Oxygen Glucose Deprivation (OGD). The panels are chemiluminescent images taken using a Biorad ChemiDoc^TM^ MP imaging system, which provides information of the molecular weight/size of the bands (weights depicted to left of blots). The red boxes indicate the bands featured in Fig 2 a,d. The signals of the bands from the original, unprocessed immunoblots were measured using using Biorad Image Lab software v6.0.1. Note, the brightness and contrast of the representative blots in Fig 2 a,d has been enhanced for visualisation purposes only.

*
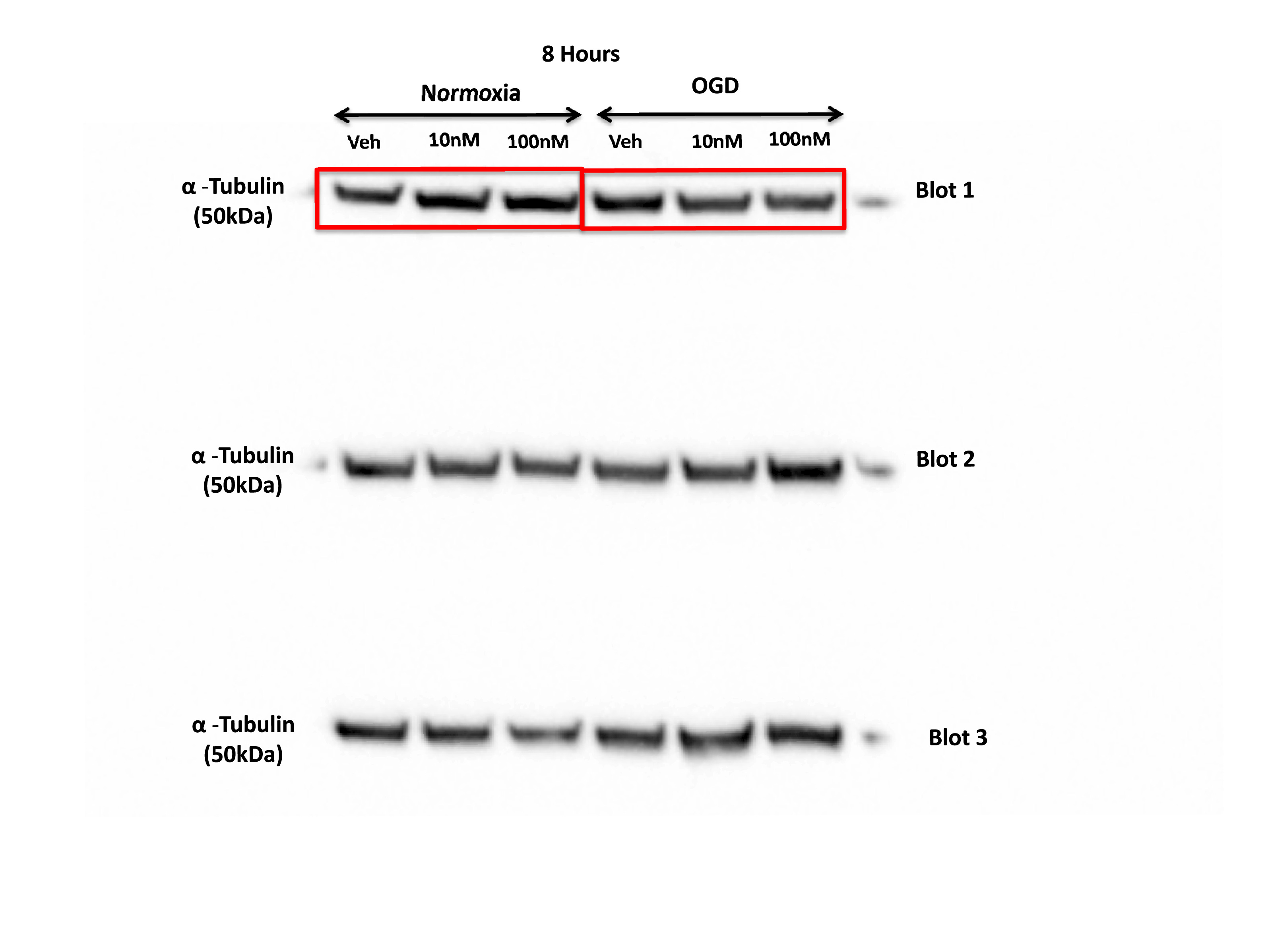
*

**Supplementary Fig 9** Full unedited blots for Supplementary Fig 3 a,d– used for the analysis of alpha-tubulin in pericytes exposed to 8 hours of normoxia or Oxygen Glucose Deprivation (OGD). The panels are chemiluminescent images taken using a Biorad ChemiDoc^TM^ MP imaging system, which provides information of the molecular weight/size of the bands (weights depicted to left of blots). The red boxes indicate the bands featured in Fig 2 a,d. The signals of the bands from the original, unprocessed immunoblots were measured using using Biorad Image Lab software v6.0.1. Note, the brightness and contrast of the representative blots in Fig 2 a,d has been enhanced for visualisation purposes only.

***
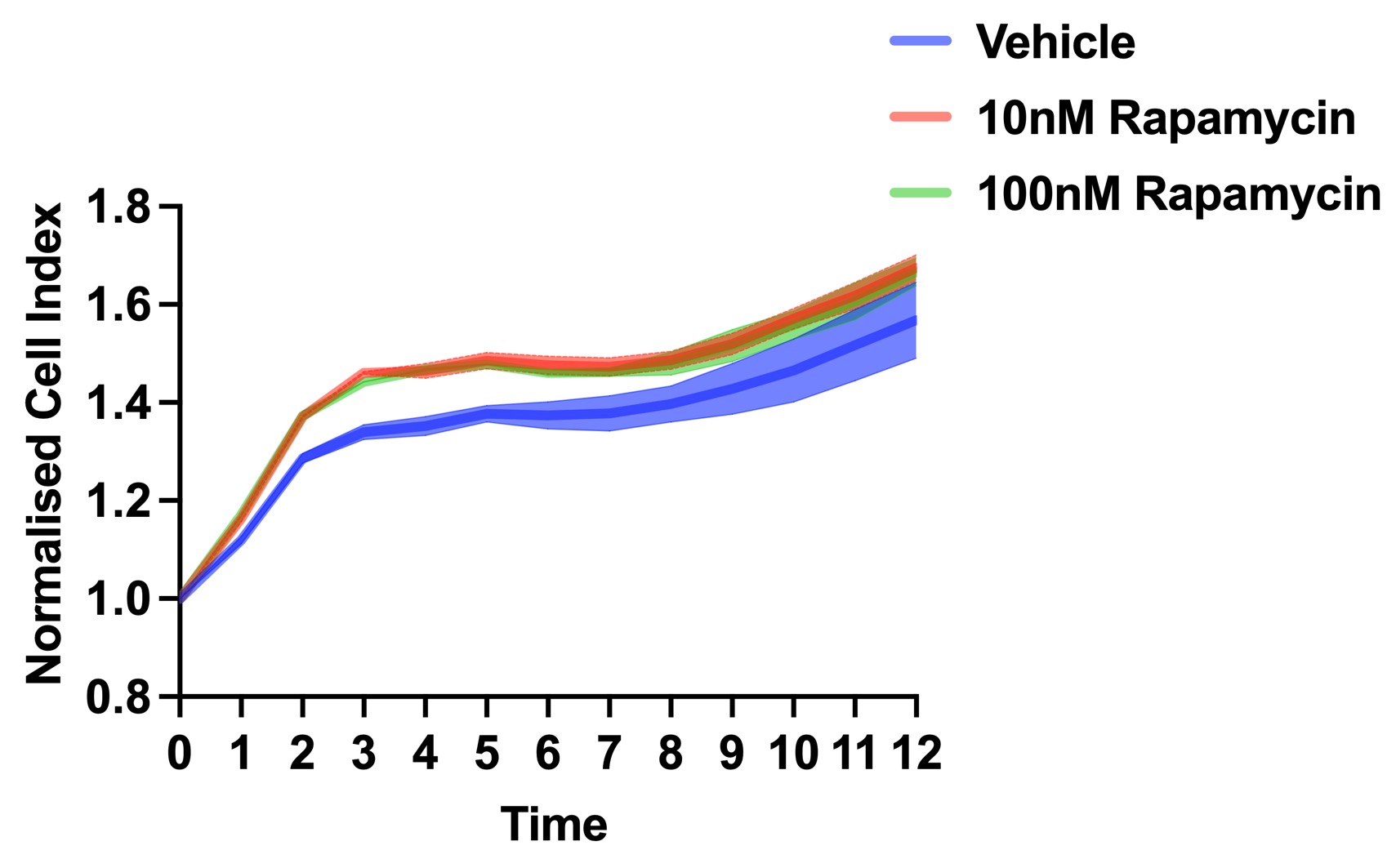
***

**Supplementary Fig 10** Pericyte contractility during Normoxia. Average normalized cell index for vehicle (black), 10nM rapamycin (light blue) and 100nM rapamycin (dark blue) treated cells during 12 h of normoxia. n = 2 per treatment group.

 
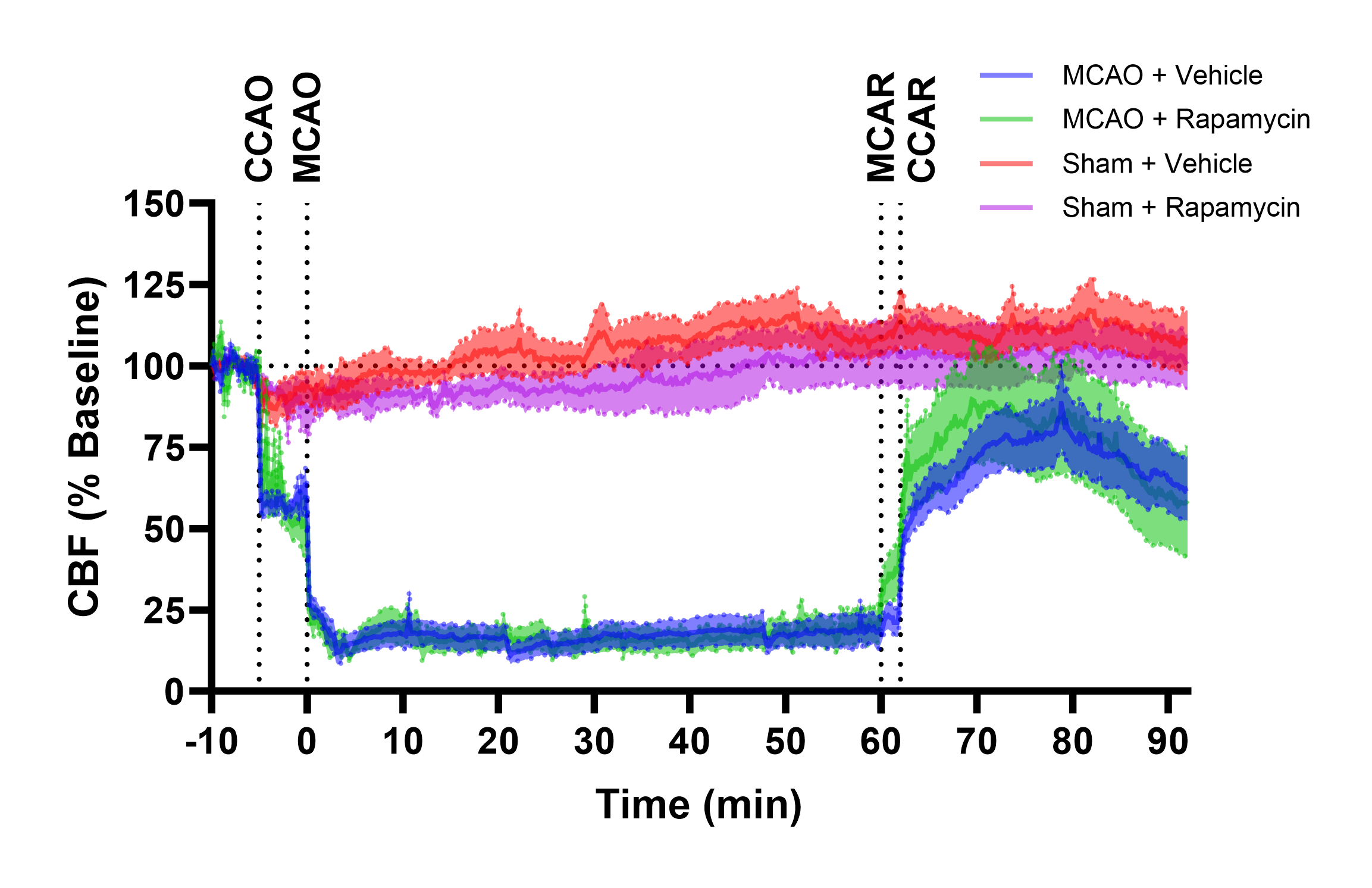
**Supplementary Fig 11** Rapamycin does not affect cerebral blood flow throughout the MCAO and reperfusion periods in the upper somatosensory cortex. Laser-Doppler trace representing cerebral blood flow for NG2-DsRed mice undergoing right-side middle cerebral artery occlusion (MCAO) surgery. Dark lines represent mean CBF and shaded area represents SEM for each treatment group. Number of individual mice analysed for each group: MCAO + vehicle (blue), N=7; MCAO + rapamycin (green), N=8; sham + vehicle (red), N=5; sham + rapamycin (magenta), N=4. Y-axis represents cerebral blood flow (CBF) normalised to a 5-minute section of baseline. X-axis represents time (minutes) normalised to the commencement of MCAO. Horizontal dotted line represents baseline CBF (100%). Vertical dotted lines and corresponding text represent the following: CCAO = time when common carotid artery occlusion occurred; MCAO = time when middle cerebral artery occlusion occurred; MCAR = time when middle cerebral artery recanalisation occurred; CCAR = time when common carotid artery recanalisation occurred.

**References**

1. Abbott NJ, Dolman DE, Drndarski S, Fredriksson SM. An improved in vitro blood-brain barrier model: rat brain endothelial cells co-cultured with astrocytes. Methods Mol Biol. 2012;814:415-30. doi: 10.1007/978-1-61779-452-0_28.

2. Redzic ZB, Rabie T, Sutherland BA, Buchan AM. Differential effects of paracrine factors on the survival of cells of the neurovascular unit during oxygen glucose deprivation. Int J Stroke. 2015;10(3):407-14. doi: 10.1111/ijs.12197.

3. Morris GP, Wright AL, Tan RP, Gladbach A, Ittner LM, Vissel B. A Comparative Study of Variables Influencing Ischemic Injury in the Longa and Koizumi Methods of Intraluminal Filament Middle Cerebral Artery Occlusion in Mice. PLoS One. 2016;11(2):e0148503. doi: 10.1371/journal.pone.0148503.

4. Winkler EA, Bell RD, Zlokovic BV. Pericyte-specific expression of PDGF beta receptor in mouse models with normal and deficient PDGF beta receptor signaling. Mol Neurodegener. 2010;5:32. doi: 10.1186/1750-1326-5-32.

5. Smyth LCD, Rustenhoven J, Scotter EL, Schweder P, Faull RLM, Park TIH, et al. Markers for human brain pericytes and smooth muscle cells. J Chem Neuroanat. 2018;92:48-60. doi: 10.1016/j.jchemneu.2018.06.001.
